# Biochemical profiling of diabetes disease progression by multivariate vibrational microspectroscopy of the pancreas

**DOI:** 10.1038/s41598-017-07015-z

**Published:** 2017-07-27

**Authors:** Christoffer Nord, Maria Eriksson, Andrea Dicker, Anna Eriksson, Eivind Grong, Erwin Ilegems, Ronald Mårvik, Bård Kulseng, Per-Olof Berggren, András Gorzsás, Ulf Ahlgren

**Affiliations:** 1 Umeå Centre for Molecular Medicine, Umeå University, Umeå, Sweden; 2 The Rolf Luft Research Center for Diabetes and Endocrinology, Karolinska Institutet, Stockholm, Sweden; 3 Centre for Obesity, Department of Surgery, St. Olavs Hospital, Trondheim University Hospital, Trondheim, Norway; 4 Obesity Research Group, Department of Cancer Research and Molecular Medicine, Faculty of Medicine, Norwegian University of Science and Technology, Trondheim, Norway; 5 Department of Chemistry, Umeå University, Umeå, Sweden

## Abstract

Despite the dramatic increase in the prevalence of diabetes, techniques for *in situ* studies of the underlying pancreatic biochemistry are lacking. Such methods would facilitate obtaining mechanistic understanding of diabetes pathophysiology and aid in prognostic and/or diagnostic assessments. In this report we demonstrate how a multivariate imaging approach (orthogonal projections to latent structures - discriminant analysis) can be applied to generate full vibrational microspectroscopic profiles of pancreatic tissues. These profiles enable extraction of known and previously unrecorded biochemical alterations in models of diabetes, and allow for classification of the investigated tissue with regards to tissue type, strain and stage of disease progression. Most significantly, the approach provided evidence for dramatic alterations of the pancreatic biochemistry at the initial onset of immune-infiltration in the Non Obese Diabetic model for type 1 diabetes. Further, it enabled detection of a previously undocumented accumulation of collagen fibrils in the leptin deficient *ob/ob* mouse islets. By generating high quality spectral profiles through the tissue capsule of hydrated human pancreata and by *in vivo* Raman imaging of pancreatic islets transplanted to the anterior chamber of the eye, we provide critical feasibility studies for the translation of this technique to diagnostic assessments of pancreatic biochemistry *in vivo*.

## Introduction

The number of individuals inflicted with diabetes globally is expected to rise from 415 to over 640 million by 2040^1^. Yet, the availability of diagnostic tools for biochemical characterization of the pancreas in both Type 1 and Type 2 diabetes (T1D and T2D, respectively) is limited. Since routine diagnostic biopsy of pancreatic tissue in patients is not feasible^2^ (or at least questionable), essentially only indirect means exist to study pancreatic status *in vivo* (e.g. by analyses of circulating biomarkers or peripheral tissues). Significant efforts are currently being directed at establishing approaches for non-invasive imaging of the pancreas in relation to diabetes, e.g. using MRI or SPECT^3, 4^. However, due to low spatial resolution and their dependency on the measurement of a (single) contrast agent interacting with its target (e.g. a cell surface receptor), these techniques have only limited potential to study the complex biochemical alterations that occur during disease progression^4^. Metabolomics and proteomics approaches can provide abundant *ex vivo* biochemical information, but untargeted approaches using these techniques are difficult (i.e. *a priori* knowledge is often required)^5^. In addition their spatial context is limited. Whereas techniques for laser capture microscopy of pancreatic tissue can enrich the amount of a specific cell type in pancreatic tissue preparations for further analytical studies^6^, they provide limited sample sizes and are not directly applicable *in vivo*.

Vibrational microspectroscopy (VMS) techniques, including complementary Fourier Transform Infrared (FT-IR) and Raman microspectroscopy, provide powerful ways to gain insight into the biochemical composition of tissues for biomedical applications^7^. VMS probes the complex chemical composition of tissues based on functional groups and molecular structures/symmetries (including e.g. alpha-helix/beta-sheet alterations of proteins^8^). Chemical maps at spatial resolutions in the micrometer (wavelength dependent diffraction limited FT-IR systems^9^) to nanometer range (confocal Raman systems, augmented techniques) can be routinely generated based on unique vibrational spectral signatures with no requirement for external imaging agents. These spectral signatures can be considered as fingerprints of the molecules present in the chemical matrix. Moreover, VMS provides a tool for non-destructive *in situ* measurements. Since pathological anomalies are commonly associated with tissue specific changes in biochemical composition, VMS techniques have (given the features outlined above) been used as a sensitive chemotyping tool to detect phenotypic markers of diseases^10^. A significant challenge in VMS-based profiling of biomedical tissues however is the inherent complexity of the spectral data obtained, together with variations due to biodiversity and the presence of non-diagnostic spectral features. The common approach to handle this complexity is to address only pre-selected chemical changes based on *a priori* knowledge or hypothesis, relying on the tracking of an arbitrary number of diagnostic bands, to proceed with targeted analysis without complete chemical profiling. Alternatively, multivariate methods can be employed for data mining, either unguided (such as by principal component analysis, PCA) or guided (such as by constrained multivariate curve resolution methods (MCR), or by discriminant analysis techniques).

Since PCA aims to find the largest variation in the dataset, it is often suboptimal for separating classes (such as disease stages, etc.) and the resulting principal components usually represent a mixture of factors, including even physical properties, and not only chemical compositional changes^11^. MCR based methods, on the other hand, attempt to decompose the complex spectra in the dataset to a set of pure component profiles. This is a very popular approach as it results in easier interpretation using concentration and spectral profiles, as opposed to PCA, which uses abstract mathematical representations in the forms of Scores and Loadings, based on a combination of factors^12^. However, the resolved “pure” component profiles may not represent pure chemical compounds in the case of biomedical samples and are often insufficient for differentiating classes on their own. Thus, MCR frequently needs to be followed by a classification method (such as cluster analysis, image segmentation, etc.)^12^. In contrast, discriminant analysis techniques are designed to differentiate pre-defined classes in the dataset in a single step. In this study we have assessed the potential of orthogonal projections to latent structures discriminant analysis (OPLS-DA)^11, 13^ to analyse VMS data from healthy and diseased tissues and organs, more specifically the pancreas in settings of diabetes. OPLS-DA is a recent member of the discriminant analysis family gaining popularity in metabolomics and plant sciences, in part due to its ability to effectively differentiate sources of variation in a dataset^11^, thus allowing focus to be directed towards the separation of user-defined classes (e.g. cell-types, genotypes, disease stages etc.) and away from other types of variation (e.g. biodiversity and experimental variation^14^). The resulting predictive components (PCs) describe the differences between the user-defined classes, whilst orthogonal components (OCs) describe variation within those classes^11^.

**Figure 1:**
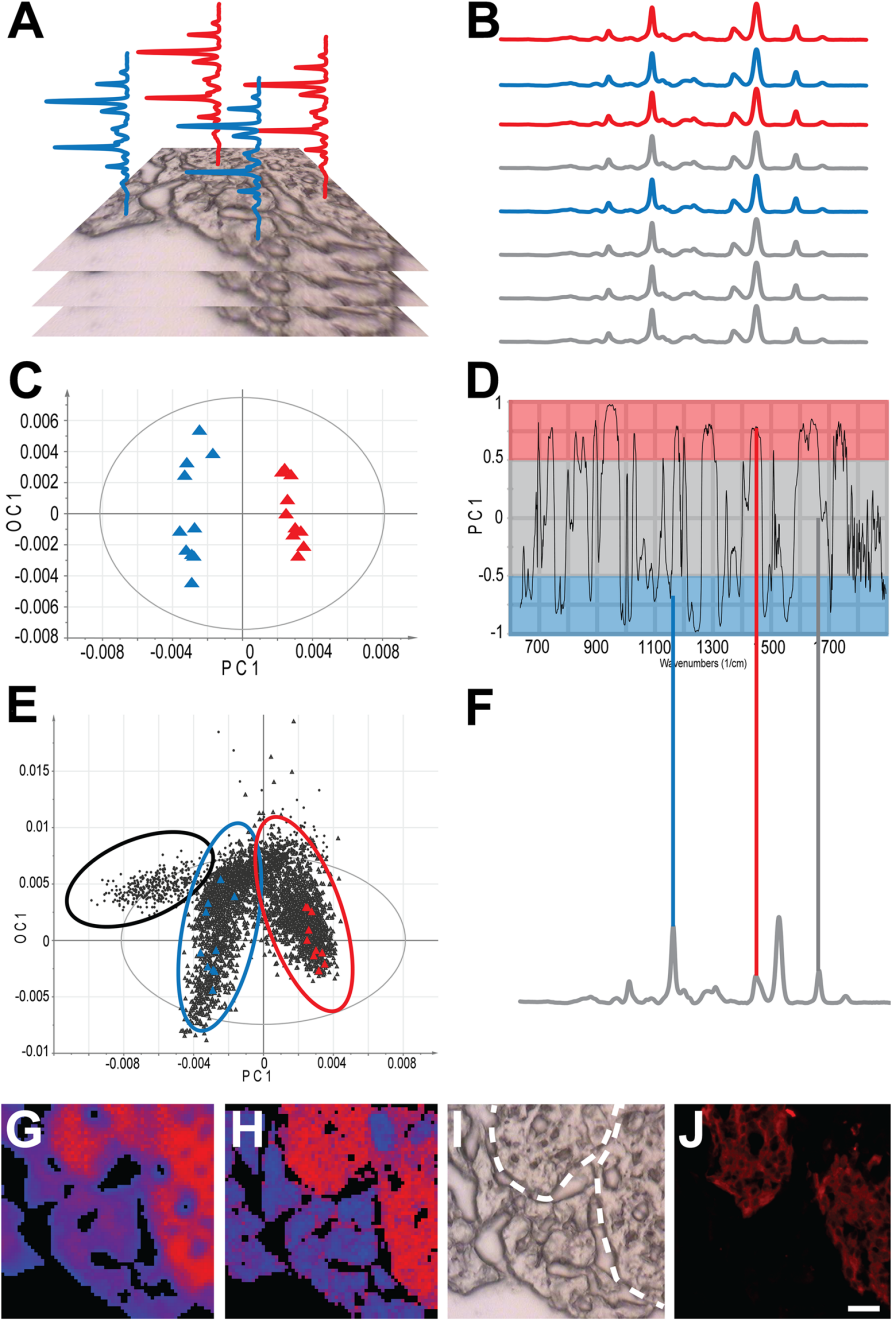
Schematic representation of the analysis workflow. (**A**) Vibrational spectra are collected from a set of samples with each recorded image containing several (thousands) of spectra. (**B**) Spectra of unambiguous origin are labeled according to user-defined classes (e.g. red and blue representing endocrine and exocrine tissue, respectively), with the majority of the spectra left unassigned (gray). An OPLS-DA model is constructed using only the assigned spectra. (**C**) The Scores plot visualizes the separation of the classes. (**D**) The corresponding correlation scaled Loadings describe the contribution of each spectral band to the separation. Bands on the positive side (red shade) are more intensive in the red class (endocrine tissue), bands on the negative side (blue shade) are more intensive in the blue class (exocrine tissue). Bands in the center (gray shade) do not contribute to class separation (statistically insignificant differences). (**E**) All unassigned spectra (gray) can be predicted by the model to determine their class membership, i.e. whether they belong to any of the pre-defined classes (red or blue), or none (i.e. outliers, not described by the model, representing e.g. the empty regions of the image (black)). (**F**) The Loadings plot can be correlated to the original spectra to validate the identified bands (red and blue lines mark selected diagnostic bands for the endocrine or exocrine tissue, respectively, while the gray line mark a non-diagnostic band). (**G**) Based on the Scores values, false color maps can be generated to visualize predicted class memberships. (**H**) The same process is repeated for the complementary Raman spectral dataset. False color OPLS-DA maps in (**G**) and (**H**) are derived from VMS data of C57BL/6 pancreas at 8 weeks. (**I** and **J**) The accuracy of the predictions can be validated by matching the results to the bright field image (endocrine region is marked by white dashed line (**I**) and to consecutive, stained sections (endocrine tissue is shown in red by insulin staining, (**J**). Scale bar is 25 µm for (**G**–**J**).

Although the separation is visualized by Scores plots, and the factors that contribute to this separation (i.e. the set of spectral bands that are diagnostics for the user-defined classes) can be determined via the corresponding Loadings plots, these are easier to interpret than in the case of PCA, since usually single components separate the classes, and they do not include contributions from unwanted variation (e.g. biodiversity, physical properties such as tissue thickness, etc.). Since the entire spectral range is included in the analysis, the results are based on monitoring the complete chemical profile simultaneously, and not only pre-determined targeted components. This excludes the need of *a priori* knowledge regarding molecular composition of the sample or of the suspected changes, allowing the analysis to drive the hypothesis, as opposed to the hypothesis determining the analysis. This greatly reduces bias and avoids loss of information (for schematic workflow of the data analysis procedure, see Fig. 1 **)**.

In the present work, we demonstrate how multivariate analysis (by OPLS-DA) of VMS data of the pancreas is capable of elucidating previously unrecorded biochemical alterations preceding, or taking place in conjunction with, diabetes disease progression. We further show that such data can be used for diagnostic profiling, while preserving the spatial context of the analyzed tissue. By assessments of islets of Langerhans transplanted to the anterior chamber of the eye (ACE), we prove that the technique can be used for *in vivo* monitoring of the islets, in a setup that allows for simultaneous monitoring of the islets complete biochemical composition together with aspects of islet function. Lastly, we address the feasibility of translating the approach to human studies.

## Results

### OPLS-DA analysis of VMS data enables classification of disease models and identification of biochemical alterations unique to diabetes pathology

To validate our method and evaluate its diagnostic potential, we first addressed the possibility to identify chemical differences between the endocrine and exocrine areas of the pancreas. We sectioned pancreas from healthy C57BL/6 mice and subjected the same unstained tissue sections to two independent, but complementary, VMS techniques: FT-IR and Raman microspectroscopy. OPLS-DA analysis of the resulting hyperspectral image data clearly delineated the endocrine and exocrine components of the tissues (Fig. 1G–J). Likewise, in a more complicated setting that includes the presence of auto immune cell types in type 1 diabetes (T1D), infiltrating CD3^+^ T-lymphocytes could be delineated by analyses of the Non Obese Diabetic (NOD) mouse model^15, 16^ (Fig. S1A–H). Thus, OPLS-DA allows for label-free classification of endocrine, exocrine and infiltrating cell types in the pancreas based on the VMS spectral profiles that reflects their chemical composition. After successfully differentiating the primary pancreatic cell types, we further validated the OPLS-DA approach in a defined setting. We recorded and analyzed VMS data from the pancreas of transgenic RIP-HAT mice, expressing human islet amyloid polypeptide (h-IAPP) specifically in the islet β-cells. Similarly to what has been observed in human T2D patients, IAPP in these mice aggregates to form cytotoxic amyloid deposits^17^. OPLS-DA was able to identify the same spectral band shift as has been previously assigned to amyloid deposits^18^ (Fig. S2), thereby validating the accuracy of the methodology and underlining its diagnostic potential. With these results at hand, we assessed the potential of the approach for untargeted biochemical profiling of model systems of T1D and T2D.

### VMS profiling of *ob/ob* pancreata

**Figure 2:**
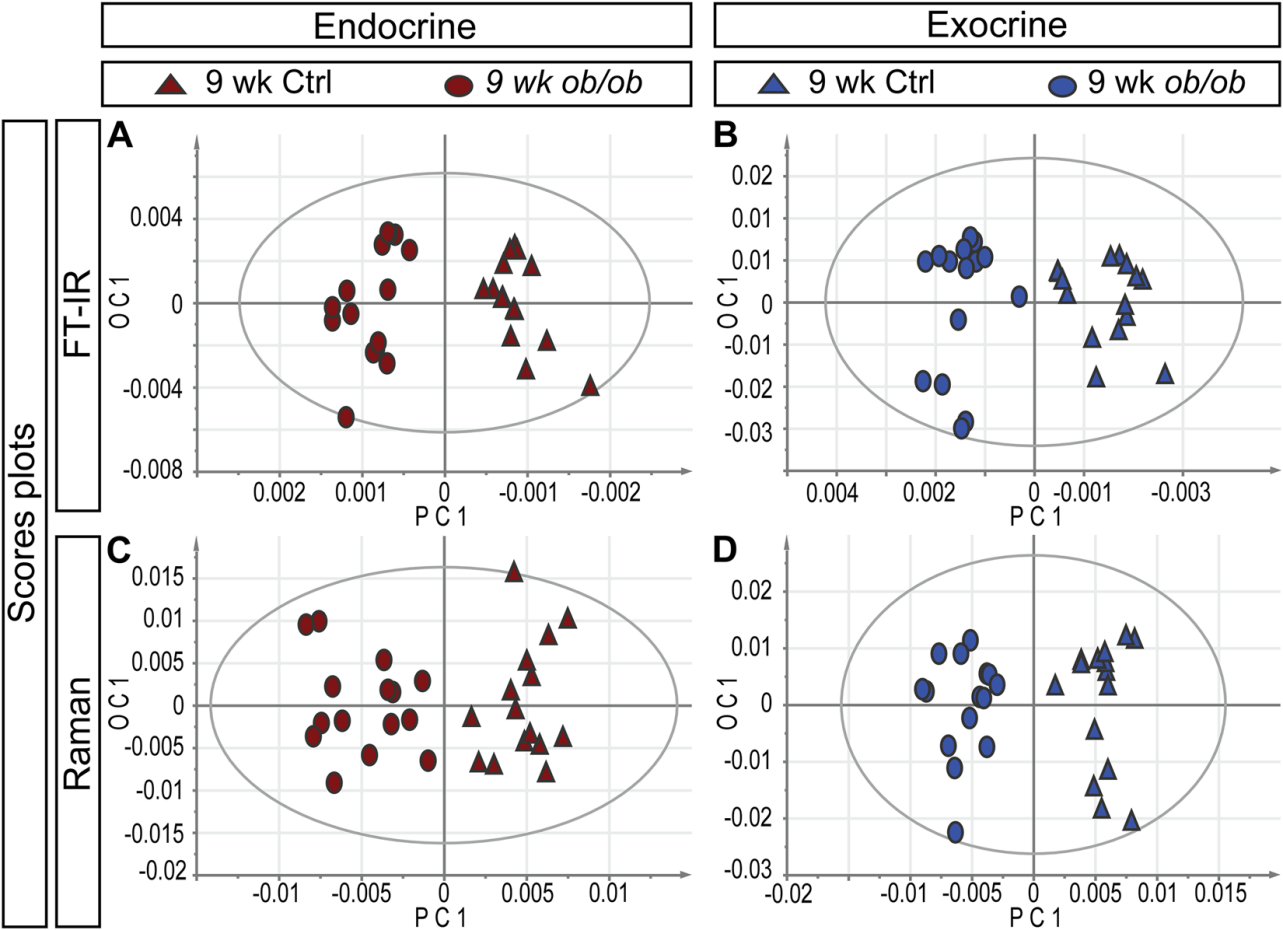
Classification of *ob/ob* and control mice mice by FTIR and Raman spectral profiles. (**A–D**) OPLS-DA Scores plots based on FT-IR (**A**,**B**) and Raman (**C**,**D**) spectra from endocrine (**A**,**C**) and exocrine (**B**,**D**) regions respectively, at 9 weeks of age. Circles (ο) represent *ob/ob* mice and triangles (Δ) represent +*/*? control mice. The ellipses in (**A**–**D**) corresponds to the 95% confidence interval of the model (Hotellings T2). For the corresponding correlation scaled OPLS-DA Loadings, see Fig. S3A–D. For a detailed list of spectral (biochemical) changes, see Table S1.

The leptin-deficient *ob/ob* mouse is widely used to study aspects of obesity and insulin resistance, and has been put forward as a model to study early stages of T2D^19^. We performed OPLS-DA analyses of VMS data obtained from *ob/ob* and of control (+/*− or* +/+) pancreata at 3 weeks (pre-obese) and 9 weeks (obese) of age. The *ob/ob* and lean control groups were clearly separated at 9 weeks of age based on the spectral profiles of both the endocrine and exocrine tissue (Fig. 2, Fig. S3A–D). The observed biochemical changes in the *ob/ob* endocrine tissue at 9 weeks of age could be assigned to a higher proportion of β-sheet structures, a higher proportion of lipids, and an increase in the proportion of collagen as compared to control. Other changes included a lower proportion of carbohydrates and a lower concentration of nucleic acids per area unit (for a detailed list of spectral changes in the *ob/ob* model, see Table S1). While the exocrine tissue of *ob/ob* and control mice could also be differentiated based on their spectral profiles (Fig. 2B and D), the biochemical differences were somewhat less pronounced (c.f. Q2 (cum) values in Table S3). In this case, separating factors were similar but not identical to those of the endocrine tissue (Table S1). When including data from 3 weeks old animals, the separation between the groups became less pronounced in both tissues (Fig. S4 and Movie S1), indicating that the chemical differences between the groups increased with age (c.f. Q2(cum) values in Table S3).

### VMS profiling of Non Obese Diabetic (NOD) mice

**Figure 3:**
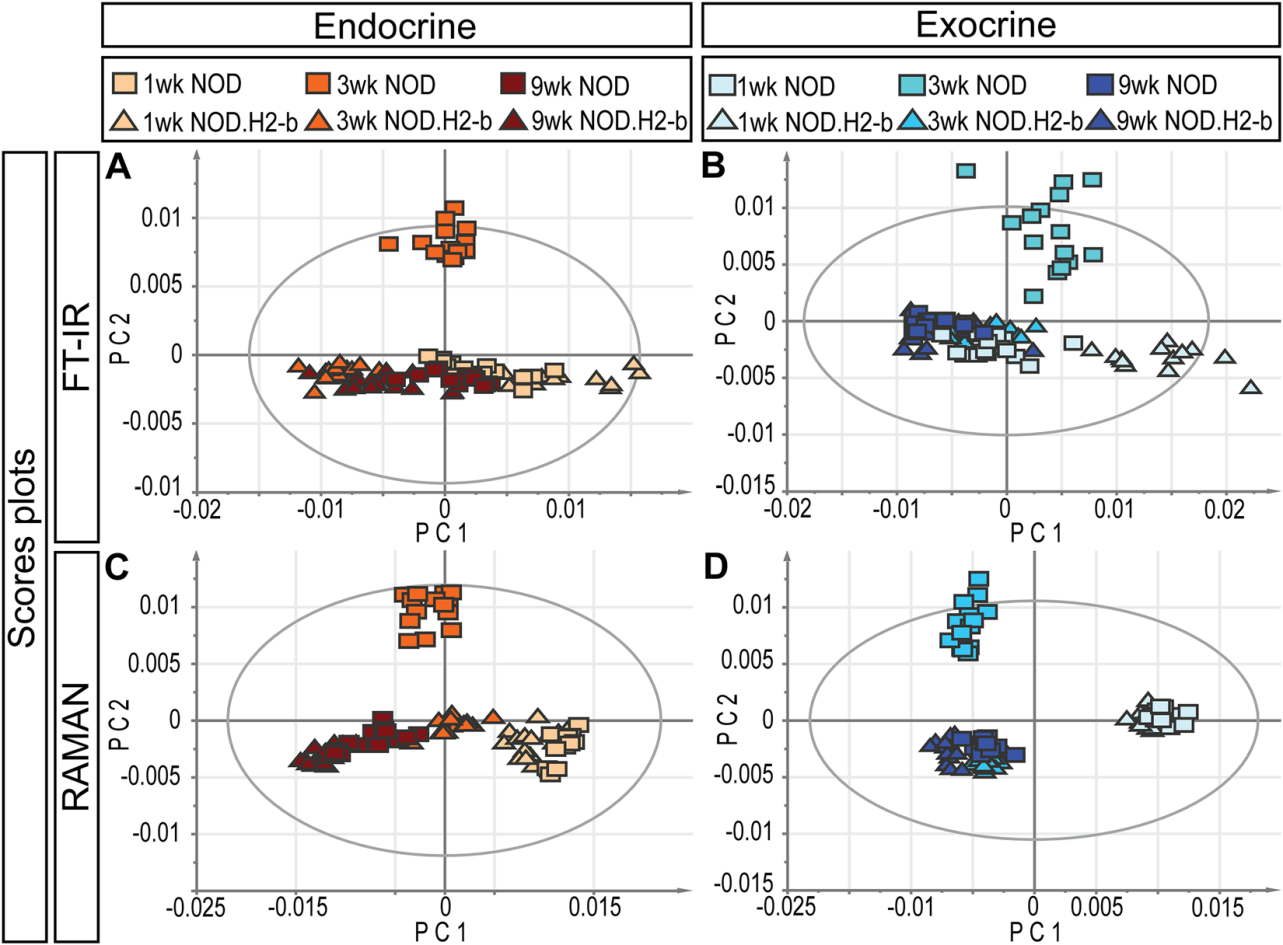
Classification of NOD and NOD.H2-b control mice by FTIR and Raman spectral profiles. OPLS-DA Scores plots based on FT-IR (**A**,**B**) and Raman (**C**,**D**) spectra from endocrine (**A**,**C**) and exocrine (**B**,**D**) regions, respectively. Squares (☐) represent NOD mice and triangles (Δ) represent NOD.H2-b control mice. The NOD endocrine and exocrine component display a unique chemical composition at 3 weeks of age. The ellipses in (**A**–**D**) corresponds to the 95% confidence interval of the model (Hotellings T2). For the corresponding correlation scaled OPLS-DA Loadings, see Fig. S3E–H. For a detailed list of spectral (biochemical) changes, see Table S2.

In the NOD model for T1D, the development of clinical diabetes is preceded by an inflammation of the pancreatic islets (insulitis), which mediates β-cell destruction. Immune infiltration is first detected around 3 weeks of age, whereas hyperglycemia usually is not diagnosed until after 10 weeks^15, 16^. VMS data was collected from tissue sections of NOD and non-diabetes prone NOD.H2-b congenic control mice at the age of 1 week (pre-insulitis), 3 weeks (around the onset of insulitis) and 9 weeks (pronounced insulitis)^20^ (see Fig. S1I–K). OPLS-DA analysis showed a more or less gradual change across the age groups that was independent of genotype (PC1 in Fig. 3). Notably, the NOD endocrine and exocrine component both displayed a unique chemical composition at 3 weeks of age, i.e. around the onset of insulitis (PC2 in Fig. 3, Table S2, Movie S2 and Fig. S3E–H). At this stage, our data indicate a proportional increase in band intensities assigned to collagen and DNA, accompanied by a corresponding proportional decrease in carbohydrates in the exocrine tissue of NOD compared to control mice (Table S2). The endocrine component of 3-week old NOD mice displayed different, yet specific characteristics: a decreased proportion of proteins, coupled to overall changes in their secondary structures indicating lower proportions of helical/random coil structures (Table S2). While it is most likely that these changes originate from a decrease in the amounts of proteins with helical structures, contributions from an increased presence of β-sheet proteins cannot be fully excluded.

Jointly, the results obtained from *ob/ob* and NOD mice demonstrate that OPLS-DA modelling of VMS data may be used to extract biochemical hallmark profiles from pancreatic tissues of T1D and T2D models that reflect the genotype, age and stage of disease progression of individual samples.

### Raman imaging of the anterior chamber of the eye (RACE) enables biochemical characterization of islets of Langerhans *in vivo*

**Figure 4:**
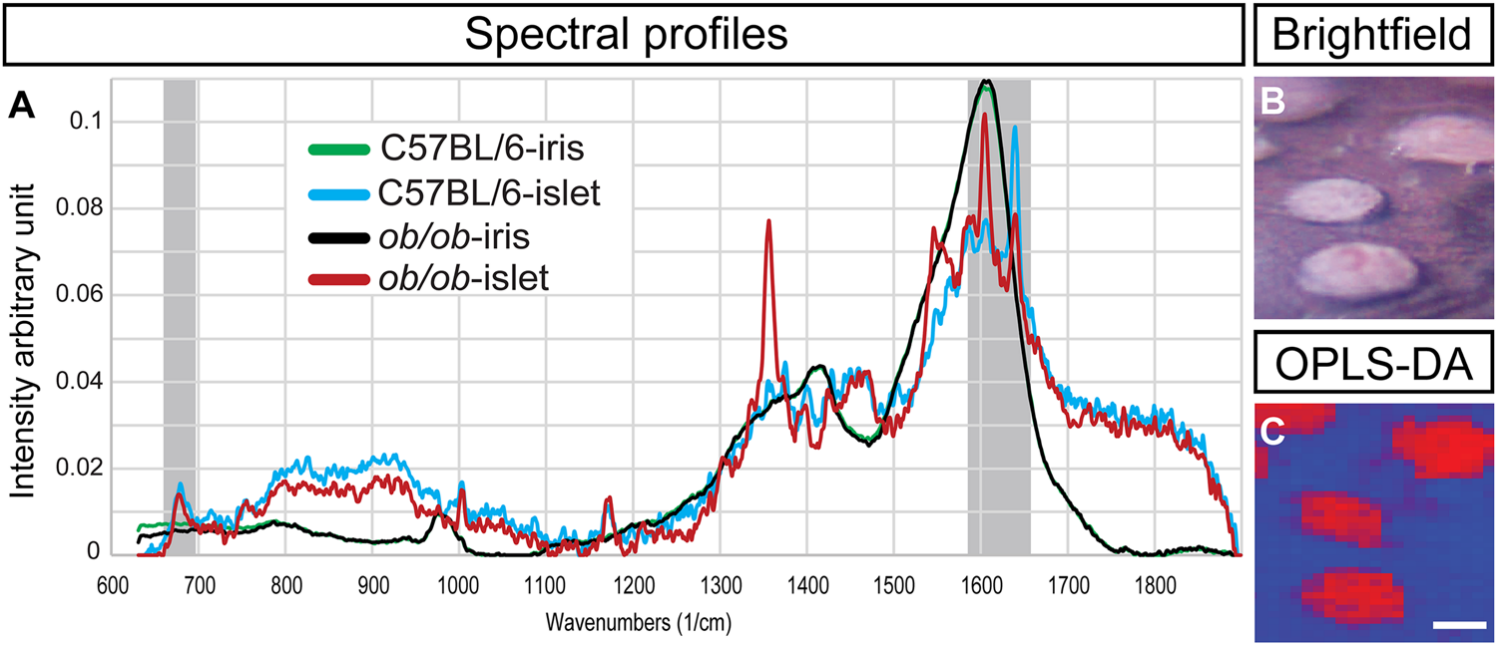
Raman imaging of the anterior chamber of the eye (RACE) enables biochemical characterization of the islets of Langerhans *in vivo*. (**A**) Representative Raman spectral profiles resolved via Multivariate Curve Resolution – Alternating Least Squares (MCR-ALS)^12^ from islets syngeneically transplanted into the anterior chamber of the eye of a mouse, 4 weeks post engraftment (black: *ob/ob*, green: *C57BL/6* control), and from the transplanted islets (red: *ob/ob*, blue: *C57BL/6* control). Two regions are marked by gray shading, indicating some of the bands identified by the OPLS-DA model that are characteristic of islets: -S-S- vibrations (around 673 cm^−1^) previously assigned to insulin, and amide I vibrations (centered around 1600 cm^−1^), sensitive to alterations in protein composition/structure. (**B**) Representative bright field image of the iris of the host containing grafted islets. (**C**) OPLS-DA false color image corresponding to (**B**), based on complete Raman spectral profiles, i.e. on differences in chemical composition. Blue: iris of host; red: transplanted islets. Scale bar is 100 µm for (**B** and **C**). Model details are listed in Table S3, and spectra are provided as Supplementary Dataset 1.

Using the cornea as a natural body window and the anterior chamber of the eye (ACE) as a transplantation site, longitudinal assessments of various aspects of β-cell biology have been studied *in vivo* by confocal microscopy^21–23^. Using the same principle, we evaluated the potential of multivariate Raman imaging to investigate the biochemical profile of islets *in vivo* using C57BL/6 and *ob/ob* mice engrafted with syngeneic islets in an open Raman microspectroscopy setup. OPLS-DA analysis clearly identified islets based on their chemical composition. Spectral profiles, resolved selectively from islets and the surrounding eye tissue via Multivariate Curve Resolution – Alternating Least Squares (MCR-ALS), could then be compared between strains (Fig. 4). Non-islet spectra were found to be identical, demonstrating the lack of chemical alterations in non-islet tissue. However, islet spectra showed markedly different profiles between strains. As expected, spectra recorded by RACE were not identical to those obtained from dry sections, particularly with respect to spectral bandwidths. While this limits direct comparisons, important similarities can be identified, indicating analogous chemical compositional changes, e.g. in protein secondary structures (increased β-sheet proportions in *ob/ob* islets compared to control). Notably, a band previously assigned to insulin^24^ could also be observed in the islet spectra (Fig. 4A). This demonstrates that the technique can be applied to monitor chemical compositional changes of islets *in vivo*, with the potential to simultaneously monitor their function in the same experiment.

### Raman imaging enables extraction of high quality spectral profiles through the tissue capsule of mouse and human pancreata

**Figure 5:**
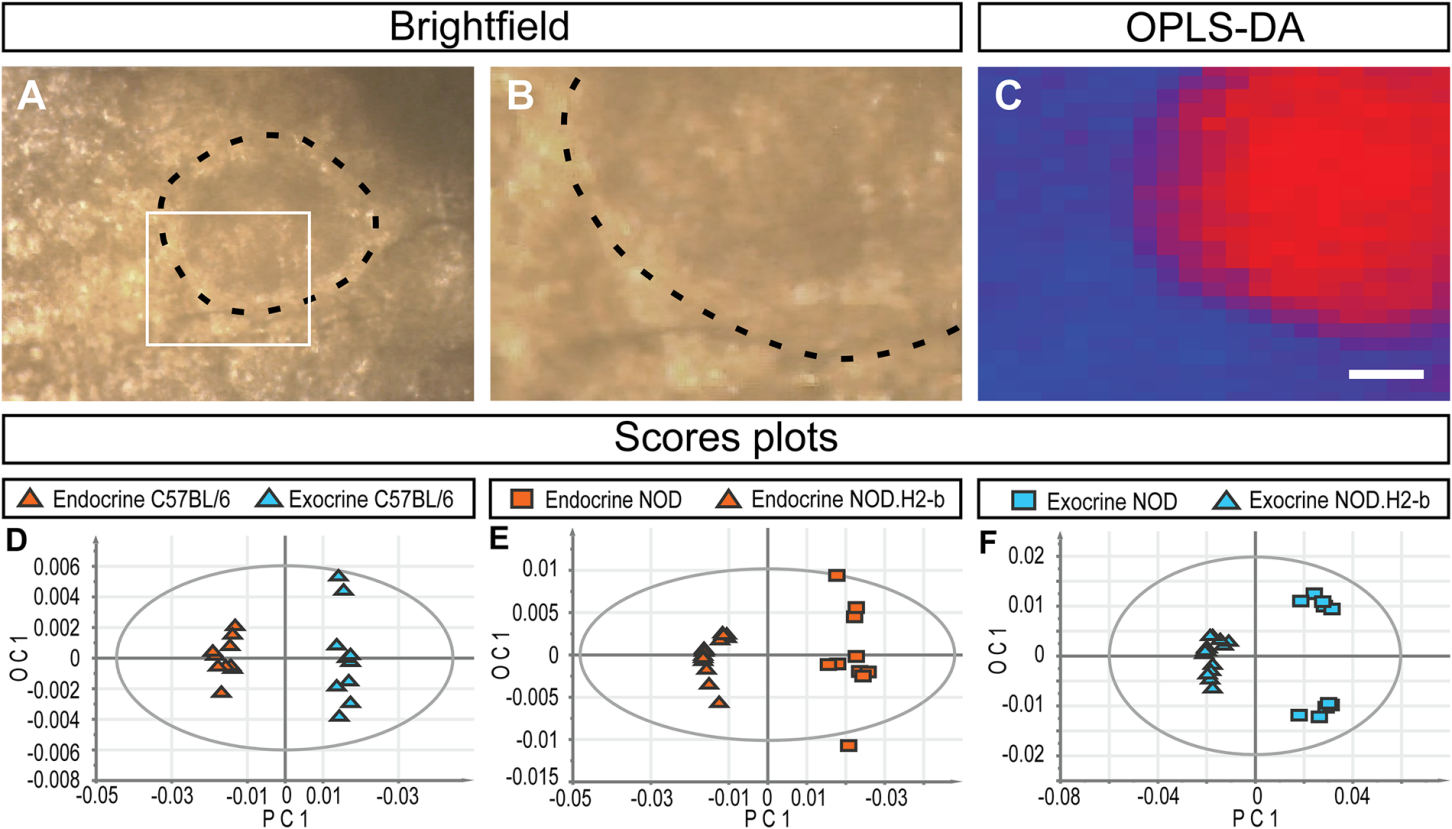
Classification of diabetes disease models through the intact pancreatic tissue capsule by OPLS-DA analysis of Raman spectra. (**A** and **B**) Bright field images of the intact pancreas of an 8 weeks old C57BL/6 mouse. The broken black line marks an islet located close to the surface and the area indicated by the white box in (**A**) is shown magnified in (**B**). (**C**) OPLS-DA false color image corresponding to (**B**), based on complete chemical composition (Raman spectral profiles), without external agents (staining, labels, etc.). Blue: exocrine tissue; red: endocrine tissue. (**D**) OPLS-DA Scores plot based on Raman spectra of selected pixels from endocrine (red triangles) and exocrine (blue triangles) regions of C57BL/6 mice. (**E** and **F**) OPLS-DA Scores plots based on Raman spectra of selected pixels from endocrine (red, **E**) and exocrine (blue, **F**) tissue, respectively, of intact NOD pancreata at 4 weeks (squares) and congenic NOD.H2-b control (triangles) pancreata at the same age. Raman spectra obtained from outside the pancreas, through the connective tissue capsule, allowing OPLS-DA analysis to separate the models based on assessments of both endocrine and exocrine tissue regions. The ellipses in (**D**,**E** and **F**) correspond to the 95% confidence interval of the model (Hotellings T2). Scale bar in (**C**) is 17 µm for (**B** and **C**) and 50 in µm (**A**). Model details are listed in Table S3, and spectra are provided as Supplementary Dataset 1.

**Figure 6:**
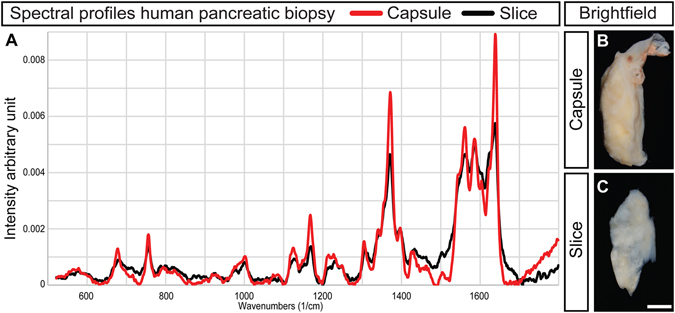
Raman imaging through the connective tissue capsule of human pancreata enables rapid, non-damaging collection of high quality spectra. (**A**) representative Raman spectra obtained through the tissue capsule (red line) and from the exposed tissue surface (black line) of resected human pancreas. Their matching profiles indicate that information about the human pancreatic biochemistry may be recorded at a small distance from the organ without the need to penetrate the tissue capsule, pointing to the feasibility of human studies analogous to the mice models. (**B**) Bright field image of the resected pancreas from which Raman spectra was recorded through the tissue capsule. (**C**) Tissue slice from (**B**), used to record Raman spectra from an exposed pancreas tissue surface. Scale bar in C is 500 µm for (**B** and **C**).

*In vivo* applications of Raman microspectroscopy are beginning to emerge, employing specially designed setups to detect spectral differences in tissues and correlate these with pathological conditions^25^. These applications require that Raman spectra of sufficient quality can be rapidly recorded without incurring tissue damage. Based on our results obtained from dry sections and transplanted islets *in vivo*, we investigated the potential for translating our OPLS-DA based approach to diagnostics of the pancreas *in situ*. We subjected intact pancreatic lobes from mice to Raman imaging using an open microspectroscopy setup to perform imaging through the connective tissue capsule. Although high quality spectral profiles could be generated from freshly isolated pancreatic tissue (data not shown), the pancreas, removed from its blood supply, rapidly degrades *in vitro*. To avoid potential tissue degradation influencing the results, we performed experiments on paraformaldehyde (PFA) fixed pancreata. From a data analysis perspective, the complexity of such spectra is similar to those obtained from an *in vivo* setting. Our method identified unique spectral profiles for exocrine tissue and islets in the intact splenic lobe of C57BL/6 mice (Fig. 5A–D). When applying the same experimental approach to intact splenic lobes from NOD and NODH2.b mice at 4 weeks of age, we could generate diagnostic spectral profiles differentiating diabetes prone individuals from congenic controls (Fig. 5E and F). Notably, when recording through the tissue capsule, we could identify these individuals even when basing the assessment exclusively on the exocrine tissue (Fig. 5F). In a similar manner we investigated the potential translation of the approach to studies of the human pancreas (Fig. 6). We could rapidly generate high quality spectral profiles from morphologically normal, PFA-fixed human pancreatic tissues, using low levels of laser excitation at a small distance from the connective tissue capsule. By superimposing spectra obtained from the cut transection margin, we could verify that the obtained spectra primarily reflected pancreatic tissue and not the connective tissue capsule.

Jointly, these results demonstrate that both murine and human pancreas are well suited for OPLS-DA based Raman diagnostics, indicating the potential of the approach for biochemical profiling of the intact organ *in vivo* using e.g. custom built endoscopic setups.

## Discussion

With emphasis on the pancreas, we investigated the utility of OPLS-DA based evaluation of VMS data as a novel approach to gather biochemical information about disease pathogenesis of cell types and organs *in situ*. The presented method combines the sensitive, high spatial resolution, label-free recording of biochemical profiles via VMS with powerful multivariate analysis via OPLS-DA, which utilizes the entire spectral information, as opposed to tracking pre-selected chemicals. The approach enables the identification of sources of variation, thereby providing easy means of classification and visualisation based on user defined categories (e.g. cell types, genotype, stage, etc.). This leads to easier automation (including the potential of self-learning) and interpretation (as opposed to e.g. PCA, which often leaves variations mixed). We validated the method by directly analysing the pancreas in the complicated settings of diabetes (using NOD and *ob/ob* mouse models for T1D and T2D, respectively), as this provides a significant challenge both for the collection and the analysis of the data, characteristic for studies of disease pathologies. Hereby, chemical compositional and structural changes could be derived using a single method.

It should be noted that the sensitivity of vibrational spectroscopy could vary greatly depending on the method used and the nature of the compound. While specialised cases can push the sensitivity to reach single molecule(!) detection, the presented setup can be considered to operate in the micromolar concentration range for the vast majority of compounds in the samples of the present study. Similarly, the specificity of vibrational spectroscopy is generally good for classes of compounds, although it can be considerably harder to identify a certain member of a class (e.g. a particular protein among all proteins). Specificity can be further compromised by overlapping bands and the overall chemical matrix of the samples. In our case, dried, PFA fixed and native (i.e. non fixed) samples all have their unique chemical (and optophysical) backgrounds, potentially influencing spectral properties and interpretations. Indeed differences in spectral shapes and positions were observed when comparing dried samples to wet ones, but we could not detect a corresponding loss in specificity.

Our study indicates that already portrayed alterations in the pancreas can be detected at earlier stages than previously reported. Accumulation of IAPP has been documented by radioimmunoassay in *ob/ob* mice from week 16^26^. Unlike human IAPP, rodent IAPP does not form amyloid deposits, but it does form beta-sheet structures^27^. We could observe an increase in the relative abundance of β-sheet proteins in the *ob/ob* endocrine tissue even before week 9, potentially reflecting this process. The higher proportion of lipids observed in the *ob/ob* endocrine tissue agrees with islet β-cell lipodeposition^28^, and the decreased concentration of DNA per area unit of the islet may be attributed to β-cell hypertrophy^22^. However, the observed increase in collagen proportions in *ob/ob* islet tissue has not been reported previously. The *ob/ob* islet vasculature has been shown to adapt to increased insulin demand and vessel hypertension by increased capillary size^29^. It appears reasonable that collagen accumulation around the islet capillaries contributes to this adaptation, since collagen accumulation is a documented feature of vessel hypertension^30^. To verify this notion, we performed electron microscopy investigations, which could confirm a significant accumulation of collagen fibrils around the islet blood vessels of *ob/ob* islets and an increase in the thickness of collagen-containing vascular basement membranes as compared to control mice (Fig. S5).

In the NOD case, we find the spectral signatures derived at 3 weeks to be of particular interest. The unique differences in biochemical composition of NOD pancreata at this early stage might be unexpected as this precedes, or coincides with, the first signs of CD3^+^ T-cell infiltration of the tissue (see Fig. S1). Although a recent study suggested that increased immune cell infiltration of the exocrine pancreas could contribute to the pathogenesis of T1D^31^, based on the methodologies employed (OPT and VMS) and the age of the mice, the alterations we report are unlikely to originate from direct contributions of infiltrating T-cells. Instead, they are likely to reflect causative processes related to very early aspects of the initial stages of the disease. For instance, increased production of interferon-α by plasmacytoid dendritic cells has been recently detected in NOD islets exclusively at 3 weeks of age^32^. While our results suggest that also the exocrine tissue is affected, further studies are required to dissect the significance of this observation and how it can be coupled to underlying molecular mechanisms.

Although the precise nature of some of the biochemical alterations reported here remains to be elucidated, particularly with respect to mechanistic implications, they could serve as useful diagnostic fingerprints and a resource for the research community (see Tables S1 and S2). As such, they may be used to inform about both stage and disease specific features, which could be used as prognostic or diagnostic tools. They may also provide a compass for more detailed targeted analyses using complementary approaches by which specific biochemical compounds or molecular mechanisms could be identified. Our results show that similar studies should be possible to conduct on bio-banked material, including wax-embedded human autopsy material.

The opportunity to simultaneously monitor the full range of biochemical changes by the proposed approach opens up for a variety of pre-clinical, and potentially clinical, research assessments that may be difficult (or even impossible) to perform by other existing techniques. This includes the possibility to broadly study functional responses at the biochemical level subject to stimuli in the form of e.g. drug administration, infections or dietary changes. Based on our results, this should be possible in diabetes research by Raman microspectroscopy based assessments of islets engrafted into the anterior chamber of the eye (RACE). In this case, parallel confocal imaging may enable longitudinal studies of the dynamics of biochemical alterations, while at the same time obtaining information of the corresponding islet pathophysiology. Similarly, the technique should be possible to combine with optical coherence microscopy (OCM) techniques to provide simultaneous information about aspects of islet vascularization and β-cell destruction of islets grafted to the ACE^33, 34^. Further, confocal and OCM based setups have been used for real time intravital imaging of the exteriorized pancreas, and an extension of our approach to this kind of setup is directly feasible^34–36^.

Combined with our demonstrations of the possibility to generate full spectral profiles through the tissue capsule of the intact gland, these results point to a potential for direct *in vivo* monitoring of the pancreatic biochemistry. Although still in developmental stage, clinical applications of Raman microspectroscopy are beginning to emerge, utilizing e.g. fiber optic probes^37, 38^. Such devices, when tailored for endoscopic Raman imaging of the pancreas combined with tuned multivariate analysis (e.g. OPLS-DA) could be developed as powerful tools to discern biochemical information about disease progression, with minimal surgical invasion and without the need for organ penetration and external agents (labels, dyes etc.).

Although the focus of our study has been within the area of diabetes, the presented method may well find its utility in assessments of other pathological conditions. In studies of the pancreas, this may include pancreatitis or pancreatic cancers. However, the method is by no means limited to the pancreas, and could be applied to the biochemical profiling of other organs or tissues. As such, it may constitute a versatile addition to the toolbox available for studying disease pathogenesis. Similarly to a recent approach for cerebral glioma surgery^39^, Raman imaging using fiber optic probes could also aid in various types of resective surgeries by biochemically differentiating healthy and diseased tissues. The key to success relies in meeting data analysis challenges with powerful, fast and reliable multivariate methods that can be tuned to focus on the question at hand and filter unrelated variations. Jointly, our results demonstrate the utility of multivariate vibrational microspectroscopy as a tool for assessing the biochemical landscape of the pancreas.

## Materials and Methods

### Animals and tissue isolation

Female *ob/ob* ^*Umeå*^ and control *(i.e*. +/+ *or* +/*ob)* littermates were obtained from local breeding colonies at Umeå University and Karolinska Institutet. Female NOD (NOD/ShiLt*J*), NOD.H2-b (NOD.B10Sn-*H* ^*2b*^ */J*) and C57BL/6 *J* were obtained from local breeding colonies at Umeå University and Karolinska Institutet or purchased from Charles River (Germany). RIP-HAT (FVB/N-Tg (Ins2-IAPP)RHFSoel/*J*) mice were obtained from a local breeding colony at Umeå University. Mice were sacrificed by cervical dislocation, the pancreata isolated and fixed in 4% paraformaldehyde (Sigma-Aldrich, USA) for 2.5 h. The samples were washed 2 × 30 min in PBS and the respective splenic, gastric and duodenal pancreatic lobes^40^ were separated for further processing. The animal experiments described in this study were approved by the Ethical Committee on Animal Experiments for Northern Sweden and performed according to Karolinska Institutet’s guidelines for the care and use of animals in research and approved by the institute’s Animal Ethics Committee.

### Tissue processing, immunohistochemistry and OPT imaging

Pancreatic tissue for cryosectioning were equilibrated in 30% sucrose (Sigma-Aldrich, USA) and frozen in O.C.T (Tissue-Tek, Fisher Scientific, USA) using Cryomolds (Tissue-Tek, Fisher Scientific, USA) in −80 °C. Samples were cryosectioned with a section thickness of 8 µm onto CaF_2_ slides (Crystan Limited, UK) for VMS imaging and consecutive sections for immunohistochemistry were collected on Superfrost slides (Thermo Fisher Scientific, USA). Sections on CaF_2_ slides were briefly rinsed in PBS and placed in a desiccator for at least 48 h prior to FTIR and Raman microspectroscopic imaging. The consecutive slides for immunohistochemistry were prewashed in PBS 3 × 5 min and blocked for 40 min using a blocking solution containing 10% fetal calf serum (Sigma-Aldrich, USA) and 0.01% sodium azide (NaN_3_, Sigma-Aldrich, USA) in TBST. The sections were incubated ON with guinea pig α-inulin (DAKO, Sweden), washed as before and incubated with Alexa-595 α-guinea pig (Thermo Fisher Scientific, USA) for 2 h. NOD and NODh2.b mice were additionally labelled with rabbit α-CD3 (Sigma-Aldrich, USA) and Alexa-488 α-rabbit (Thermo Fisher Scientific, USA). The sections were subsequently washed and dehydrated for at least 48 h. The latter step was performed to obtain a dehydrated morphology similar to the sections on CaF_2_ slides, when serving as a reference for VMS assessments. Thioflavin S labelling for detection of amyloid deposits was performed as previously described^41^. All sections were photographed using a Nikon Eclipse E800 microscope equipped with plan fluor 10/0.30 and 20x/0.50 lenses and a Nikon DS-Ri1 digital camera (Nikon, Japan). For Raman imaging of the intact pancreas, the pancreas was isolated, fixed for 2.5 h and washed in PBS before imaging. For imaging of fresh tissue, the pancreas was isolated, washed briefly in PBS and thereafter immediately imaged by Raman microspectroscopy. Tissues for OPT imaging were processed and analyzed as previously described^42, 43^.

### Transmission electron microscopy (TEM)

Pancreata were perfused with fixative reagent (2,5% glutaraldehyde + 1% paraformaldehyde in 0.1 M phosphate buffer, pH 7.4), dissected and small pieces were further incubated at room temperature for 30 min and overnight at 4 °C in fixative. Specimens were rinsed in 0.1 M phosphate buffer, pH 7.4 and postfixed in 2% osmium tetroxide 0.1 M phosphate buffer, pH 7.4 at 4 °C for two hours, dehydrated in ethanol followed by acetone and embedded in LX-112 (Ladd, Burlington, Vermont, USA). Semi-thin sections were cut and stained with toluidine blue O and used for light microscopic analysis. Ultrathin sections (approximately 50–60 nm) were cut by a Leica EM UC 6 (Leica, Wien, Austria) and contrasted with uranyl acetate followed by lead citrate and examined in a Hitachi HT 7700 transmission electron microscope (Tokyo, Japan) at 80 kV. Digital images were taken by using a Veleta camera (Olympus Soft Imaging Solutions, GmbH, Münster, Germany).

### Human samples

Biopsies from human pancreatic tissue were obtained from patients undergoing open distal pancreatectomy due to neuroendocrine tumors of the pancreatic tail. The surgical procedure was performed according to standardized hospital routine. Following surgery, the resected specimen was immediately evaluated by a consultant pathologist and frozen sections from the distal transection margin was collected for analysis. In all patients the transection margin was confirmed tumor-free. A 0.5–1.0 cm^3^ block of morphologically normal pancreatic tissue was excised from the transection margin by the pathologist, ensuring that further pathological diagnostics was not affected. The biopsies were immediately immersed in 4% PFA. The experimentation on human pancreatic tissue was conducted according to the guidelines laid down in the Declaration of Helsinki and was approved by the Regional Ethics Committee, Northern-Norway. Written informed consent was obtained from all participants. Ethical approval for human samples was obtained prior to the experiment (2015/1054 REK Northern Norway). Written, informed consent was collected after thorough information from a senior consultant surgeon.

### FTIR Microspectroscopy

Spectra were recorded in transmission mode on a Bruker Tensor 27 spectrometer equipped with a Hyperion 3000 microscopy accessory and a liquid N_2_ cooled 64 × 64 mercury cadmium telluride (MCT) focal plane array (FPA) detector. The entire setup was placed on a vibration-proof table. Spectra were recorded in the region 950–4000 cm^−1^, with 4 cm^−1^ spectral resolution and 32 scans co-added in double sided, forward-backward mode. FPA frame rate was 3773 Hz and integration time 0.104 ms, with offset and gain optimized for each sample between 180–230 and 0–1, respectively. Background was recorded on a clean, empty spot on the CaF_2_ carrier and automatically subtracted. Fourier transformation was carried out using a zero filling factor of 2 and Blackman-Harris 3-term apodization function. Phase correction was set to the built-in Power mode with no peak search and a phase resolution of 32. Spectra were recorded using OPUS (version 6.5 and 7, Bruker Optics GmbH, Ettlingen. Germany), cut to the fingerprint region of 950–2000 cm^−1^ and exported as.mat files for subsequent multivariate image analysis. White light images were recorded with a Sony ExwaveHAD color digital video camera mounted on the top of the microscope and exported as jpg files. Spectra used to construct the models are provided as Supplementary Dataset 1.

### Raman Microspectroscopy

For dried sections, the same material was used as for FTIR microspectroscopy, on the same infrared transparent CaF_2_ microscopy slides (Crystran Ltd., UK). Intact mouse and human pancreata were prepared as described in the respective sections and were used as is, without further treatment. Spectra were recorded using a Renishaw inVia system (Renishaw Ltd, UK), equipped with an external tunable Ar^+^-ion laser. The 514 nm laser frequency was selected by a holographic notch filter and a 2400 lines grating was used, producing a maximum spectral resolution of ca. 1 cm^−1^. Varying laser powers (1 to 100% of the total 15 mW, highest for dry sections, lowest for intact pancreata and RACE measurements) and exposure times (1 to 10 s) were applied to optimize measurements for each sample to gain the highest signal to noise ratio without sample damage. Each sample was checked for visible and spectral changes to ensure safe laser levels, i.e. no alteration during measurements. 5x to 50x magnification lenses were used, and maps were obtained using raster scanning with 1 to 50 micrometer step sizes (identical in X and Y directions) to cover the measurement area, resulting in 150–4100 spectra/image. The entire setup was placed on a vibration-proof table with a closed sample chamber, allowing only the laser used to generate the Raman signal to pass and blocking white light. Spectra were recorded in static mode, centered at 1300 cm^−1^ (resulting in a spectral region of ca. 630–1900 cm^−1^). Calibration was performed on the built-in silicon standard before every set of measurement and the laser was focused and centered using manual beam steering. 0% laser defocus was used during measurements. Spectra were recorded and processed for cosmic ray removal and multivariate noise filtering using the chemometrics package of the WiRE software (versions 3.2 and 3.4, Renishaw Ltd, UK). The resulting spectra were trimmed to the region 630–1890 cm^−1^ to eliminate PCA reconstruction artefacts affecting the highest wavenumbers before baseline correction, and saved as.txt files. White light images were recorded with a Philips USB camera connected to the system, and exported as.jpg files. Signals that could potentially originate from PFA fixation were identified in separate measurements and monitored in all recordings of fixed tissue to confirm the lack of PFA bands influencing the data analysis. Spectra used to construct the models are provided as Supplementary Dataset 1.

### Raman imaging of islets transplanted to the Anterior Chamber of the Eye (RACE)

Islets from C57BL/6 and *ob/ob* mice were harvested at 4 months and syngeneically transplanted into the ACE of recipient mice at the corresponding age as previously described^21, 23^. 4 weeks post engraftment the mice were sedated using a 1:1:2 mix of Hypnorm (VetPharma Ltd, UK), Midazolam (Hameln Phamaceuticals gmbh, Germany) and distilled water. The mice were placed on a padded tray on the microscope stand and positioned for optimal access to the eye. Raman microspectroscopy conditions were as described in the section “Raman Microspectroscopy” above, with low confocality mode selected for mapping. Spectra used to construct the models are provided as Supplementary Dataset 1.

### Multivariate Image Analysis

Spectra (.mat or.txt files) were pre-processed by an open-source software developed at the Vibrational Spectroscopy Core Facility in Umeå (http://www.kbc.umu.se/english/visp/download-visp/), written in MATLAB (versions 2013b and 2014a, Mathworks, USA), using asymmetric least squares baseline correction^44^; lambda values: 5,000 (FTIR spectra), 10,000 (Raman spectra, dry sections) and 1,000,000 (intact pancreata and RACE), p = 0.01), Savitzky-Golay smoothing (1^st^ order polynomial, with a frame number of 5) and total area normalization. In case of RACE data, Multivariate Curve Resolution – Alternating Least Squares analysis were performed in the same open-source script to obtain resolved spectral profiles corresponding to the distinct areas in the image (transplanted islet or not). Only non-negativity constraints were used for both spectra and concentration directions. Initial spectral profiles were determined by the default, SIMPLISMA based method and two components were resolved with a convergence limit of 0.1 and maximum iteration of 50. Following pre-processing, spectra were saved in.mat format and imported into SIMCA-P (version 13.0, Umetrics AB, Umeå, Sweden) for OPLS-DA. For C class models, OPLS-DA was performed using C-1 predictive components and maximum C orthogonal components on centered data. False color image maps are based on predictive component Scores, using the entire image dataset as prediction set. A MATLAB based in-house software^4^ was used for visualization (plotting). OPLS-DA model details are presented in Table S3. Spectra used to construct the models are provided as Supplementary Dataset 1.

## Electronic supplementary material

Supplementary information

Supplementary Dataset 1

Supplementary Movie 1

Supplementary Movie 2
