# Supplementary material for: Biochemical profiling of diabetes disease progression by multivariate vibrational microspectroscopy of the pancreas
Source: Sci Rep. 2017 Jul 27;7:6646. doi: 10.1038/s41598-017-07015-z (PMC5532280; doi:10.1038/s41598-017-07015-z)
Supplement: Supplementary file 1 — Supplementary information [file 41598_2017_7015_MOESM1_ESM.pdf]

Biochemical profiling of diabetes disease progression by multivariate vibrational microspectroscopy of the pancreas

Christoffer Nord[1], Maria Eriksson[1], Andrea Dicker[2], Anna Eriksson[1], Eivind Grong[3,4], Erwin Ilegems[2], Ronald Mårvik[3,4], Bård Kulseng[4], Per-Olof Berggren[2], András Gorzsás[5,*] & Ulf Ahlgren[1,*]

[1] Umeå Centre for Molecular Medicine, Umeå University, Sweden
[2] The Rolf Luft Research Center for Diabetes and Endocrinology, Karolinska Institutet, Sweden.
[3] Dept. of Gastrointestinal Surgery, St. Olavs Hospital, Trondheim University Hospital, Norway
[4] Dept. of Cancer Research and Molecular Medicine, NTNU, Norway
[5] Department of Chemistry, Umeå University, Sweden
[*] Co-senior authorship

Correspondence to Ulf.Ahlgren@umu.se

Nord et al., Fig. S1

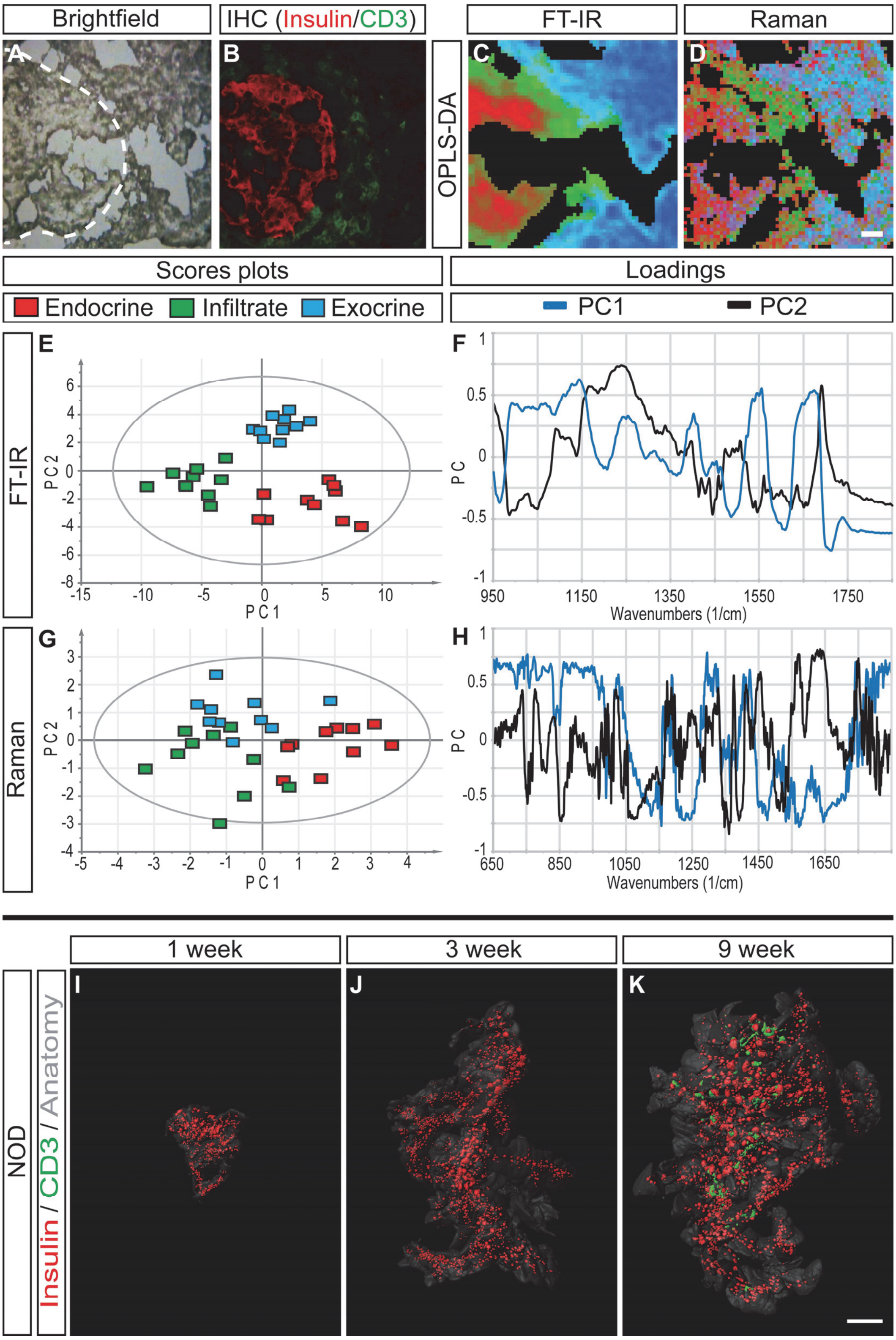

Figure S1. OPLS-DA and OPT analysis of infiltrating immune cells in the NOD model of T1D.

A, Bright field image of a pancreas section from a 9-week old NOD mouse, dried on infrared and Raman transparent CaF_2 microscopy slide. The endocrine region is marked by the white broken line. **B**, Consecutive section to (D), stained for insulin (red) and CD3 (green). **C** and **D**, OPLS-DA false color maps of the area shown in (A), based on complete FT-IR (C) and Raman (D) spectral profiles, respectively, i.e. differences in chemical composition without staining. Blue: exocrine tissue; red: endocrine tissue, green: infiltrating T-cells. **E** and **G**, OPLS-DA Scores plots based on FT-IR (E) and Raman (G) spectra, respectively, of selected pixels from endocrine (red), exocrine (blue) regions and from infiltrating T-cells cells (green), showing the separation of the classes (tissue / cell types). The ellipses in (E) and (G) correspond to the 95 % confidence interval of the model (Hotellings T2). **F** and **H**, The corresponding correlation scaled Loadings for predictive component 1 (black line) and predictive component 2 (blue line), showing FT-IR and Raman spectral bands respectively, characteristic to infiltrating auto-immune T-cells (negative bands in the black curve), and bands separating endocrine and exocrine tissue (negative and positive bands respectively, in the blue curve). The further away from the 0 value on the Y axis a band is in the negative direction in the Loadings of predictive component 1 (black line), the more characteristic it is for infiltrating CD^{3+} T-cells. The further away from the 0 value on the Y axis a band is in the negative or positive direction in the Loadings of predictive component 2 (blue line), the more characteristic it is for endocrine and exocrine cells, respectively (diagnostic bands). **I-K**, Representative iso-surface rendered OPT images of the duodenal pancreatic lobes from the NOD pancreata used for VMS analyses (in Fig. 3) at 1, 3 and 9 weeks of age. Ins^{+} islets (red) are reconstructed based on the signal from insulin-specific antibodies and infiltrating T-cells (green) are based on the signal from CD3-specific antibodies. No $CD3^{+}$ cells could be detected in the pancreas at 1 and 3 weeks of age (I and J) whereas 9-week old pancreata displayed signs of full-blown insulitis (K). Scale bar in (D) is 20 µm for A-D and scale bar in (K) is 1000 µm for I-K.

Nord et al., Fig. S2

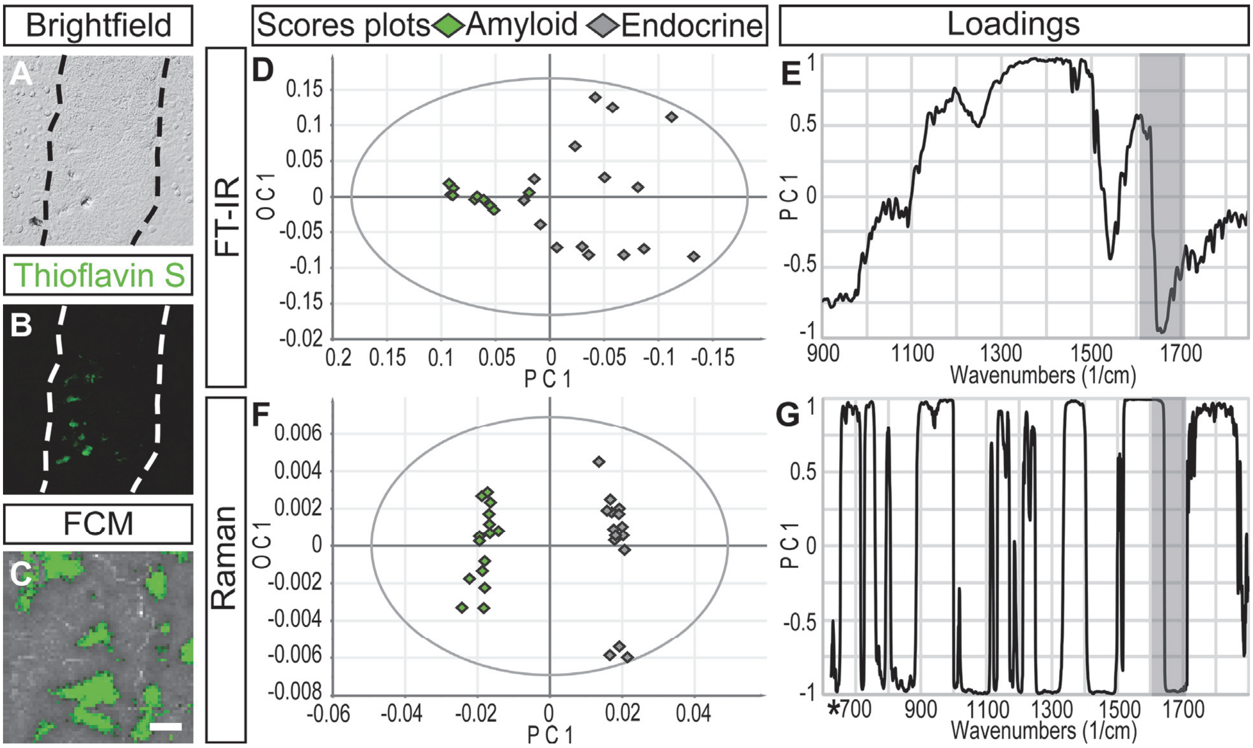

Figure S2. Detection of amyloid plaque formation by OPLS-DA analysis of VMS data from the pancreas of RIP-HAT transgenic mice.

A, Image of a pancreas section from a RIP-HAT transgenic mouse (at 23 weeks of age). The endocrine region (an islet of Langerhans) is marked by a broken line. **B**, Consecutive section to (A), stained for Thioflavin S (marker for amyloid deposits, green). **C**, OPLS-DA false color map, corresponding to the approximate area of Thioflavin S labeled cells in (B), from a consecutive unstained section based on the complete Raman spectral profile (grey: endocrine tissue; green: amyloid deposits). **D** and **F**, OPLS-DA Scores plots based on FT-IR and Raman spectra, respectively, of selected pixels from endocrine cells (grey) and amyloid deposits (green), showing their separation. The ellipses in (D) and (F) correspond to the 95 % confidence interval of the model (Hotellings T2). **E** and **G**, The corresponding correlation scaled Loadings for predictive component 1, showing FT-IR and Raman spectral bands, respectively, that separate endocrine tissue (negative) from amyloid deposits (positive). The further away from the 0 value on the Y axis a band is in the negative or positive direction, the more characteristic it is for endocrine cells and amyloid deposits respectively (diagnostic bands). The area highlighted in gray in (E) and (G) denote the amide I vibration, sensitive to protein structural changes, indicating a higher proportion of beta-sheet structures in the amyloid deposits. Asterix (*) in (G) indicates the -S-S- band previously assigned to insulin in the Raman spectra. Abbreviation; FCM, False Color Map. Scale bar in C is 50 µm in (A) and (B), and 30 µm in (C).

Nord et al., Fig. S3

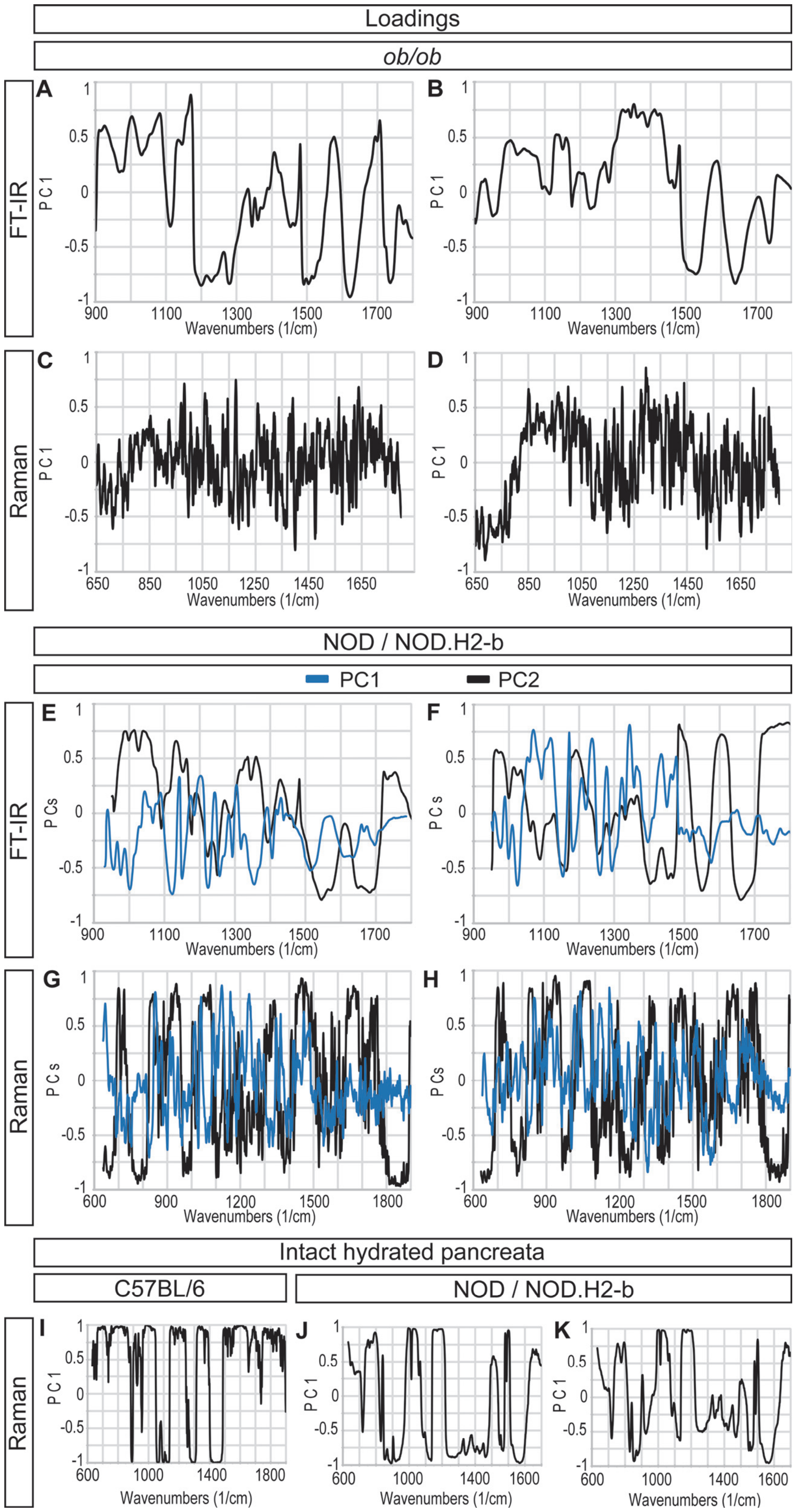

Figure S3. Correlation scaled OPLS-DA Loadings

A-D, **Correlation scaled OPLS-DA Loadings corresponding to the Scores plots in Fig. 2 A-D,** based on FT-IR (A and B) and Raman (C and D) spectra of selected pixels from endocrine (A and C) and exocrine (B and D) regions, respectively, showing the contribution of spectral bands separating *ob/ob* from control mice at 9 weeks of age. The further away from the 0 value on the Y axis a band is in the negative or positive direction, the more characteristic it is for *ob/ob* or control mice, respectively (diagnostic bands). For a detailed list of spectral (biochemical) changes, see **Table S1**. **E-H**, Correlation scaled OPLS-DA Loadings based on FT-IR (E and F) and Raman (G and H) spectra, corresponding to (Fig. 3 A-D). the Loadings for Predictive Component 1 (X axis in the Scores plot) are drawn in black and display the contribution of spectral bands corresponding to age related changes in the model, irrespective of genotype. Bands more intense in young individuals are positive and bands corresponding to old individuals are negative. Loadings for predictive component 2 (Y axis in the Scores plot) are drawn in blue, showing the contribution of spectral bands to differentiate 3 weeks old NOD pancreata (positive bands) from all other classes (negative bands). The further away from the 0 value on the Y axis a band is, the more characteristic it is for its class. For a detailed list of spectral (biochemical) changes, see **Table S2. I,** Correlation scaled Loadings for Predictive Component 1 (X axis) in Fig. 5D, showing the spectral bands differentiating endocrine (negative) and exocrine regions (positive) in normal C57BL/6 mice pancreata imaged through the connective tissue capsule. The further away from the 0 value on the Y axis a band is in the negative or positive direction, the more characteristic it is for endocrine, or exocrine regions, respectively (diagnostic bands). **J** and **K,** Correlation scaled Loadings for Predictive Component 1 (X axis in Fig 5 E and F, respectively), showing the spectral bands differentiating control NOD.H2-b (negative values) and NOD (positive values) mice, using endocrine (J) or exocrine (K) tissue regions only. The further away from the 0 value on the Y axis a band is in the negative or positive direction, the more characteristic it is for control, or NOD mice, respectively (diagnostic bands).

Nord et al., Fig. S4

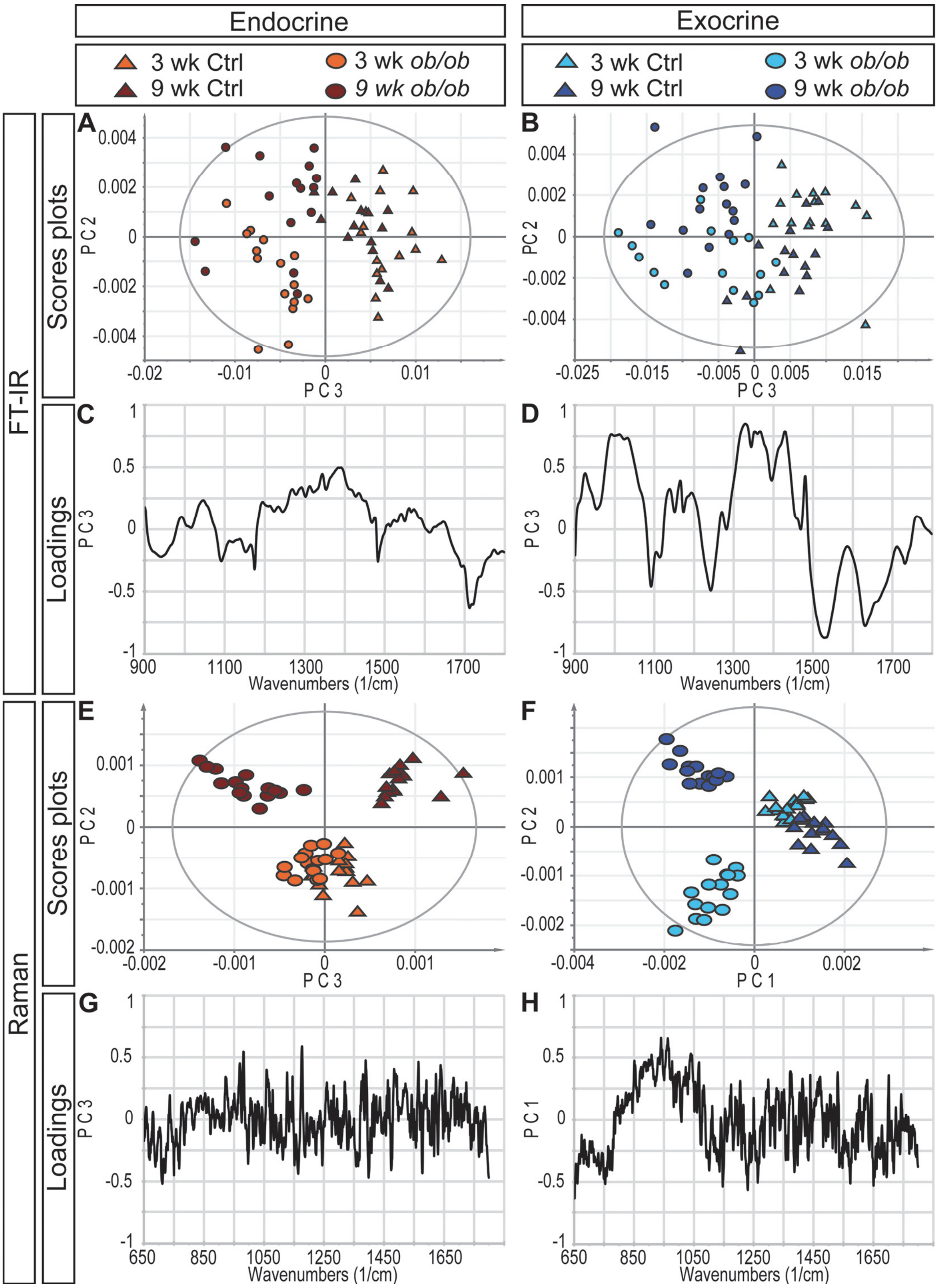

Figure S4. Classification of *ob/ob* and control mice by FT-IR and Raman spectral profiles at 3 and 9 weeks of age.

A-D, FT-IR OPLS-DA Scores (A and B) and Loadings (C and D) plots, based on spectra of selected pixels from endocrine (A and C) and exocrine (B and D) regions respectively. **E -H**, Raman OPLS-DA Scores (E and F) and Loadings (G and H) plots, based on spectra of selected pixels from endocrine (E and G) and exocrine (F and H) regions, respectively. Triangles represent control mice, circles represent *ob/ob* mice. Light shading denotes 3 weeks old individuals and dark shading 9 weeks old individuals. C, D, G and H, FT-IR and (C and D) and Raman (G and H) correlation scaled OPLS-DA Loadings corresponding to Predictive Component 3 (X axis) in (A, B and E), and predictive component 1 in (F), showing the contribution of spectral bands separating ob/ob from control mice. The further away from the 0 value on the Y axis a band is in the negative or positive direction, the more characteristic it is for *ob/ob* or control mice, respectively (diagnostic bands). The ellipses in (A, B and E, F) correspond to the 95 % confidence interval of the model (Hotellings T2). For a detailed list of spectral (biochemical) changes, see **Table S1**.

Nord et al., Fig. S5

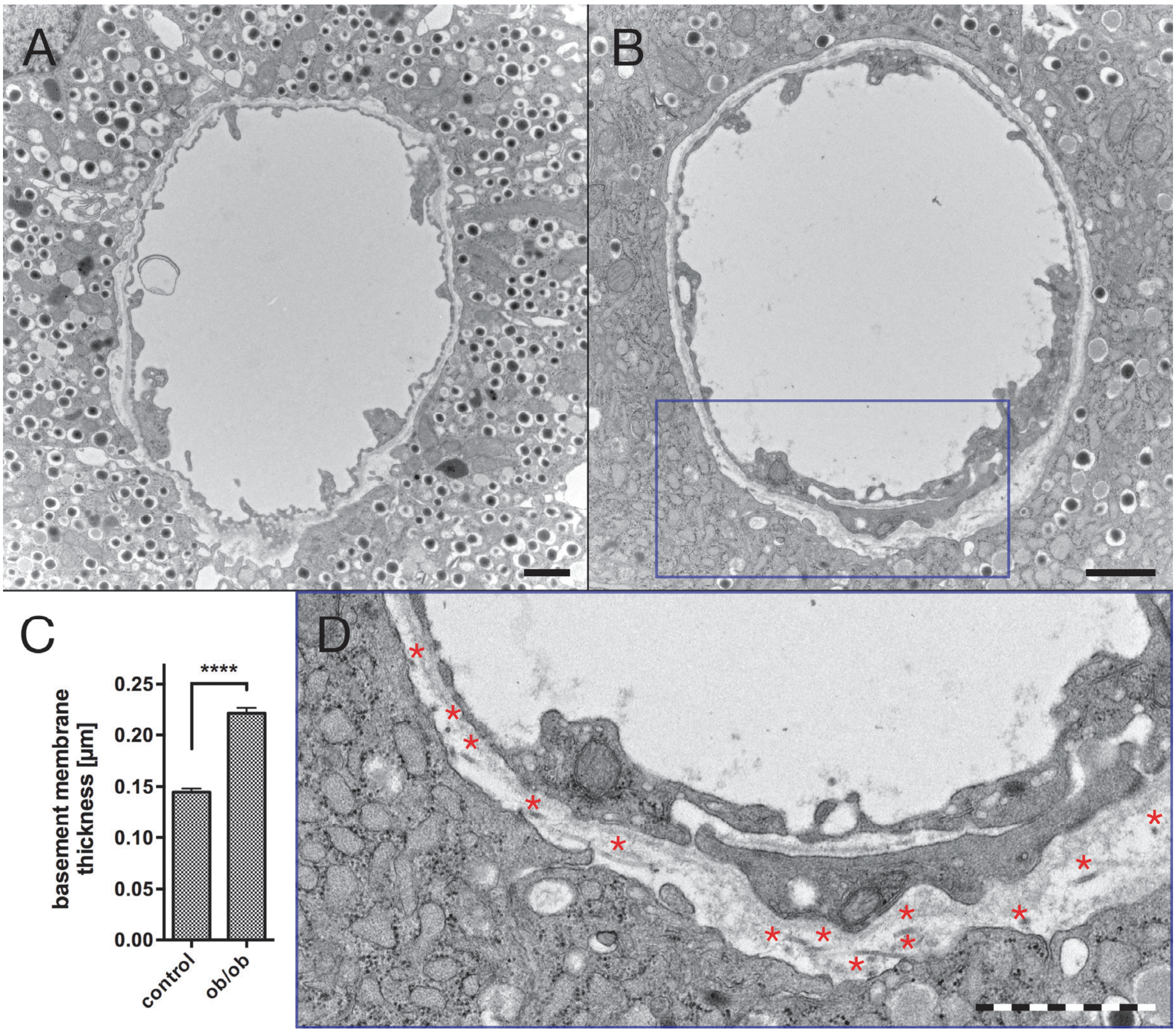

Figure S5. Collagen fibril accumulation in *ob/ob* islets.
A and **B,** Electron microscopy imaging of intra-islet blood vessels in 8-week old female mice comparing characteristics of the extracellular matrix in control lean (A) and *ob/ob* mice (B). **C**, Measurement of basement membrane thickness indicates a significant increase in *ob/ob* as compared to control (n>250 vessel segments analyzed per group, 3 mice per group). **D**, Magnified view (from blue frame in B) shows the accumulation of elongated collagen fibrils (marked by red stars) in the extracellular matrix. Error bars represent SEM. ****$P < 0.0001$. Scale bars: 1 µm.

Table S1. VMS bands separating *ob/ob* and control pancreata. Only the most prominent bands are listed (more than 50% correlation in OPLS-DA Loadings, see Fig. 2 and Fig. S4). Band positions are listed at approximate maxima or cover ranges for wide features. Relative proportions are listed as increased (+) or decreased (-) in the given class. Assignments are based on chapter chapter 8: FTIR and Raman Characteristic Frequencies in Biological Studies In: Vibrational Spectroscopy for Tissue Analysis (Eds: Rehmn, Movasaghi, Rehman), 2013, CRC Press, Taylor & Francis Group LLC, 6000 Broken Sound Parkway NW, Suite 300, Boca Raton, FL 33487-2742, USA, ISBN: 9778-1-4398-3608-8 (and references therein) and our own observations. For the endocrine tissue, separating factors are practically identical at all ages, but separation improved using data only from 9 week old individuals for FTIR spectroscopic data (Fig. 2, Table S3). For Raman spectra, separating factors show less correlation, partly due to lower signal to noise ratios. For the exocrine tissue separation improved for FTIR spectroscopic data when only spectra from 9 weeks old individuals are included (Fig. 2, Table S3), thus unique FTIR spectral bands for this model are listed separately.

Endocrine tissue

Band Position	Relative Proportion	Assignment (compounds)	Technique
660-720 cm^{-1}	+ *ob/ob* (all ages)	-C-S vibrations (in e.g. collagen, insulin), -CH and ring deformation vibrations	Raman
745 cm^{-1}	+ *ob/ob* (all ages)	Unspecific ring deformation vibrations, -C-O bending vibration	Raman
1207 cm^{-1}	+ *ob/ob* (all ages)	Amide III (proteins, collagen)	FTIR
1221 cm^{-1}	+ *ob/ob* (all ages)	Amide III (proteins, primarily beta sheet structures)	FTIR
1236 cm^{-1}	+ *ob/ob* (all ages)	-C-O, -P-O, amide III (phosphates, proteins, relatively specific to collagen)	FTIR
1280 cm^{-1}	+ *ob/ob* (all ages)	-C-O, amide III (proteins, collagen)	FTIR
1490-1550 cm^{-1}	+ *ob/ob* (all ages)	Amide II (proteins, beta sheet structures, although less specific than amide I)<br>-C=C- and -C=N (tryptophan, porphyrin, guanine, adenine)<br>=C-H (unsaturated fatty acids)	FTIR, Raman
1600-1645 cm^{-1}	+ *ob/ob* (all ages)	Amide I (proteins, mostly beta sheet structures)	FTIR, Raman
1740 cm^{-1}	+ *ob/ob* (all ages)	-C=O (lipids, phospholipids, fatty acids, esters, carboxylic acids)	FTIR
926 cm^{-1}	- *ob/ob* (all ages)	Ring vibrations (carbohydrates, nucleic acids)	FTIR
990 – 1000 cm^{-1}	- *ob/ob* (all ages)	Ring vibrations (mono-, oligo-, polysaccharides)	FTIR, Raman
1051 cm^{-1}	- *ob/ob* (all ages)	Ring vibrations, phosphates (mono-, oligo-, polysaccharides, nucleic acids)	FTIR
1072 cm^{-1}	- *ob/ob* (all ages)	Ring vibrations, phosphates (mono-, oligo-, polysaccharides, nucleic acids)	FTIR, Raman
1135 cm^{-1}	- *ob/ob* (all ages)	-C-H deformations, -C-O-C- stretching	Raman
1300-1350 cm^{-1}	- *ob/ob* (all ages)	Unspecific -C-H bending vibrations (from e.g. fatty acids / lipids, amino acids /	Raman

		proteins, DNA bases, etc)	
1360-1370 cm^{-1}	- *ob/ob* (all ages)	-C-H, -C-N vibrations, ring breathing modes of DNA bases (T, A, G), saccharides	Raman
1580-1630 cm^{-1}	- *ob/ob* (all ages)	Double bonds (C=C, C=N, C=O), in e.g. nucleic acids, proteins, olefins	Raman

Exocrine tissue

**Band Position**	**Relative Proportion**	**Assignment (compounds)**	**Technique**
1230 cm^{-1}	+ *ob/ob* (all ages)	-C-O, -P-O, amide III (phosphates, proteins, including collagen)	Raman
1490-1560 cm^{-1}	+ *ob/ob* (all ages)	Amide II (proteins)<br>-C=C- and -C=N (tryptophan, porphyrin, guanine, adenine)<br>=C-H (unsaturated fatty acids)	FTIR
1530-1570 cm^{-1}	+ *ob/ob* (all ages)		Raman
1620-1690 cm^{-1}	+ *ob/ob* (all ages)	Amide I (proteins)	FTIR, Raman
926 cm^{-1}	- *ob/ob* (all ages)	Ring vibrations (carbohydrates, nucleic acids)	FTIR, Raman
940-960 cm^{-1}	- *ob/ob* (all ages)	-C-C- and –C-H vibrations (carbohydrates, amino acids), phosphates (nucleic acids)	Raman
993 cm^{-1}	- *ob/ob* (all ages)	Ring vibrations (mono-, oligo-, polysaccharides)	FTIR, Raman
1030-1040 cm^{-1}	- *ob/ob* (all ages)	Ring vibrations, phosphates (mono-, oligo-, polysaccharides, nucleic acids)	FTIR, Raman
1490-1550 cm^{-1}	+ *ob/ob* (9 weeks of age)	Amide II (proteins)<br>-C=C- and -C=N (tryptophan, porphyrin, guanine, adenine)<br>=C-H (unsaturated fatty acids)	FTIR
1620-1670 cm^{-1}	+ *ob/ob* (9 weeks of age)	Amide I (proteins)	FTIR
1135 cm^{-1}	- *ob/ob* (9 weeks of age)	Carbohydrate ring vibrations, -C-O, -P-O (mono-, oligo-, polysaccharides, nucleic acids, lipids. Proteins and lipids can be excluded due to different amide I / II profiles)	FTIR
1305 cm^{-1}	- *ob/ob* (9 weeks of age)	-C-H in nucleic acids, amide III (proteins. Contribution less likely due to different amide I / II profiles)	FTIR
1328 cm^{-1}	- *ob/ob* (9 weeks of age)	Ring vibrations, including C-C and C-N stretches (unspecific, nucleic acids, oligo/polysaccharides, amino acids, proteins, etc.)	FTIR
1344 cm^{-1}	- *ob/ob* (9 weeks of age)	-C-H (unspecific, oligo and polysaccharides, nucleic acids, amino acids, proteins, etc.)	FTIR
1365	- *ob/ob* (9 weeks of age)	-C-H deformation (unspecific, oligo and polysaccharides, nucleic acids, amino acids, proteins, etc.)	FTIR

1398 cm^{-1}	- *ob/ob* (9 weeks of age)	-C-H (methyl- and methylene groups in proteins and nucleic acids, primarily) contributions from –COO stretch (fatty acids, amino acids) cannot be excluded	FTIR

Table S2. VMS bands separating NOD and NOD.2H-b control pancreata. Only the most prominent bands are listed (more than 50% correlation in OPLS-DA Loadings, see Figure 3). Band positions are listed at approximate maxima or cover ranges for wide features. Relative proportions are listed as increased (+) or decreased (-) in the given class. Assignments are based on chapter 8: FTIR and Raman Characteristic Frequencies in Biological Studies In: Vibrational Spectroscopy for Tissue Analysis (Eds: Rehmn, Movasaghi, Rehman), 2013, CRC Press, Taylor & Francis Group LLC, 6000 Broken Sound Parkway NW, Suite 300, Boca Raton, FL 33487-2742, USA, ISBN: 9778-1-4398-3608-8 (and references therein) and our own observations.

Endocrine tissue

Band Position	Relative Proportion	Assignment (compounds)	Technique
970 – 1066 cm^{-1}	+ with age	Carbohydrate ring vibrations (mono-, oligo-, polysaccharides, incl. glycogen)<br>-P-O (unspecific origin, e.g. phosphorylation, potential oxidative degradation of DNA)	FTIR
1135 cm^{-1}	+ with age	-C-O and ring vibrations (Carbohydrates, proteins, e.g. collagen)	FTIR
1165 cm^{-1}	+ with age	-C-O, -C-O-C- (carbohydrates, including oligo- and polysaccharides)	FTIR
1340 cm^{-1}	+ with age	-C-H (unspecific, polysaccharides, proteins, lipids)	FTIR
1365 cm^{-1}	+ with age	-C-H deformation (unspecific, proteins, lipids, carbohydrates)	FTIR
1250 cm^{-1}	- with age	Amide III (proteins)	FTIR
1520-1580 cm^{-1}	- with age	Amide II (proteins)<br>-C=C- and -C=N (tryptophan, porphyrin, guanine, adenine)	FTIR
1640-1705 cm^{-1}	- with age	Amide I (proteins, mostly helical random coil structures)	FTIR
1053-1086 cm^{-1}	+ in NOD vs control (3 weeks of age)	Carbohydrate rings (mono-, oligo-, polysaccharides)<br>Phosphate (unspecific origin, e.g. phosphorylation, potential oxidative degradation of DNA)	FTIR
1106-1133 cm^{-1}	+ in NOD vs control (3 weeks of age)	carbohydrate ring and -C-O vibrations (mono-, oligo- and polysaccharides, proteins, e.g. collagen, (phospho)lipids)	FTIR
1205 cm^{-1}	+ in NOD vs control (3 weeks of age)	-C-O, -P-O (phosphates, polysaccharides, proteins, e.g. collagen)	FTIR
1240 cm^{-1}	+ in NOD vs control (3 weeks of age)	-C-O, -P-O (phosphates in e.g. phospholipids and DNA, proteins, e.g. collagen)	FTIR
1278 cm^{-1}	+ in NOD vs control (3 weeks of age)	-C-O, amide III (proteins), although some contribution from phosphates cannot be excluded	FTIR
1321 cm^{-1}	+ in NOD vs control (3 weeks of age)	Amide III (proteins) and -C-H vibrations (unspecific, e.g. polysaccharides, proteins, nucleic acids)	FTIR

1344 cm^{-1}	+ in NOD vs control (3 weeks of age)	-C-H vibrations (unspecific, but proteins and nucleic acids in particular)	FTIR
1434 cm^{-1}	+ in NOD vs control (3 weeks of age)	-C-H (unspecific, but lipids in particular)	FTIR
1474 cm^{-1}	+ in NOD vs control (3 weeks of age)	=C-H (lipids, unsaturated fatty acids)	FTIR
1728 cm^{-1}	+ in NOD vs control (3 weeks of age)	-C=O (fatty acids, lipids, phospholipids)	FTIR
1026 cm^{-1}	- in NOD vs control (3 weeks of age)	Amide bonds, Carbohydrate ring vibrations (proteins, mono-, oligo-, polyscaccharides, incl. glycogen)	FTIR
1151 cm^{-1}	- in NOD vs control (3 weeks of age)	-C-O, -C-O-C-, -C-N contribution (carbohydrates, proteins, carotenoids)	FTIR
1254 cm^{-1}	- in NOD vs control (3 weeks of age)	Amide III (proteins)	FTIR
1396 cm^{-1}	- in NOD vs control (3 weeks of age)	-C-H (methyl- / methylene-groups in e.g. side chains of proteins)	FTIR
1516-1587 cm^{-1}	- in NOD vs control (3 weeks of age)	Amide II (proteins)<br>-C=C- and -C=N (tryptophan, porphyrin, guanine, adenine)	FTIR
1640-1703 cm^{-1}	- in NOD vs control (3 weeks of age)	Amide I (proteins, mostly helical random coil structures)	FTIR

Exocrine tissue

**Band Position**	**Relative Proportion**	**Assignment (compounds)**	**Technique**
645-665 cm^{-1}	+ with age	-C-C and -C-S vibrations, amino acids, nucleic acid bases (nucleic acids, insulin)	Raman
790 cm^{-1}	+ with age	Phosphates and ring breathing modes of nucleic acid bases (nucleic acids)	Raman
1165 cm^{-1}	+ with age	-C-O, -C-O-C- (carbohydrates, including oligo- and polysaccharides)	FTIR
1210-1260 cm^{-1}	+ with age	-C-O, -P-O, amide III (phosphates, proteins, including collagen, nucleic acids)	Raman
1360-1370 cm^{-1}	+ with age	-C-H, -C-N vibrations, ring breathing modes of DNA bases (T, A, G), saccharides	Raman
1398 cm^{-1}	+ with age	-C-H (methyl- / methylene-groups in e.g. side chains of proteins)	FTIR, Raman
1415 cm^{-1}	+ with age	-C-H (lipids)	FTIR
1452-1466 cm^{-1}	+ with age	=C-H (lipids, unsaturated fatty acids), amide III vibrations (proteins)	FTIR
1535-1570 cm^{-1}	+ with age	Amide II (proteins)<br>-C=C- and -C=N (tryptophan, porphyrin, guanine, adenine)	FTIR, Raman
1640-1701 cm^{-1}	+ with age	Amide I (proteins, mostly helical random coil structures)	FTIR
698 cm^{-1}	- with age	-C-S vibrations, -CH and ring deformation	Raman

		vibrations (amino acids, nucleic acids)	
720 cm^{-1}	- with age	-C-N vibrations (nucleotides, membrane phospholipids)	Raman
833 cm^{-1}	- with age	Phosphate and ring vibrations (phospholipids, nucleic acids, tyrosine in particular)	Raman
850 cm^{-1}	- with age	Ring stretches (carbohydrates, amino acids)	Raman
900-950 cm^{-1}	- with age	Ring vibrations (carbohydrates, nucleic acids)	Raman
960 – 990 cm^{-1}	- with age	Carbohydrate ring vibrations (mono-, oligo-, polysaccharides, incl. glycogen)<br>-P-O (unspecific origin, e.g. phosphorylation, potential oxidative degradation of DNA)	FTIR
1030-1080 cm^{-1}	- with age	Ring vibrations, phosphates (mono-, oligo-, polysaccharides, nucleic acids)	Raman
1205 cm^{-1}	- with age	Amide III, -C-O, -P-O (proteins, phosphates, including phopholipids, polysaccharides)	FTIR
1320-1346 cm^{-1}	- with age	-C-H bending vibrations (unspecific, e.g. fatty acids / lipids, amino acids / proteins, DNA bases, etc)	Raman
1480-1510 cm^{-1} (FTIR)<br>1430-1480 cm^{-1} (Raman)	- with age	Amide II (proteins)<br>-C=C- and -C=N (tryptophan, porphyrin, guanine, adenine)	FTIR, Raman
1587-1626 cm^{-1} (FTIR)<br>1640-1680 cm^{-1} (Raman)	- with age	Amide I (proteins, mostly beta sheet structures)	FTIR, Raman
1740-1760 cm^{-1}	- with age	-C=O (carboxylic acids, fatty acids, in a pH dependent fashion)	FTIR, Raman
850 cm^{-1}	+ in NOD vs control (3 weeks of age)	Ring stretches (carbohydrates, amino acids)	Raman
920 cm^{-1}	+ in NOD vs control (3 weeks of age)	Ring vibrations (carbohydrates, nucleic acids)	Raman
990 cm^{-1}	+ in NOD vs control (3 weeks of age)	Carbohydrate ring vibrations (mono-, oligo-, polysaccharides)<br>-P-O (unspecific origin, e.g. phosphorylation, potential oxidative degradation of DNA)	FTIR
1051-1086 cm^{-1}	+ in NOD vs control (3 weeks of age)	Carbohydrate ring vibrations, -P-O (mono-, oligo-, polysaccharides, nucleic acids)	FTIR, Raman
1100-1126 cm^{-1}	+ in NOD vs control (3 weeks of age)	Carbohydrate ring vibrations, -C-O, -P-O (mono-, oligo-, polysaccharides, nucleic acids, lipids. Proteins and lipids can be excluded due to different amide I / II profiles)	FTIR, Raman
1228-1246 cm^{-1}	+ in NOD vs control (3 weeks of age)	-C-O, -P-O (phosphates, nucleic acids, proteins) amide III (protein) contribution is likely to be limited due to different amide I / II profiles	FTIR
1344 cm^{-1}	+ in NOD vs control (3 weeks of age)	-C-H bending vibrations (from e.g. fatty acids / lipids, amino acids / proteins, DNA	FTIR

		bases, etc.)	
1434 cm^{-1}	+ in NOD vs control (3 weeks of age)	-C-H (unspecific, fatty acids contribution can be limited, since the corresponding –C=O bands are not increased statistically. Proteins can be excluded due to different amide I / II profiles)	FTIR
1000 cm^{-1}	- in NOD vs control (3 weeks of age)	Ring vibrations (mono-, oligo-, polysaccharides), -P-O (phosphates, nucleic acids)	Raman
1026 cm^{-1}	- in NOD vs control (3 weeks of age)	Amide bonds, Carbohydrate ring vibrations (proteins, mono-, oligo-, polyscaccharides)	FTIR
1151 cm^{-1}	- in NOD vs control (3 weeks of age)	-C-O, -C-O-C-, -C-N contribution (carbohydrates, proteins, carotenoids)	FTIR
1256 cm^{-1}	- in NOD vs control (3 weeks of age)	-P-O, -C-O, amide III (phosphates, nucleic acids, proteins)	FTIR, Raman
1300-1340 cm^{-1}	- in NOD vs control (3 weeks of age)	-C-H bending vibrations (unspecific, e.g. fatty acids / lipids, amino acids / proteins, DNA bases, etc.)	Raman
1398 cm^{-1}	- in NOD vs control (3 weeks of age)	-C-H (methyl- / methylene-groups in e.g. side chains of proteins)	FTIR
1530-1585 cm^{-1}	- in NOD vs control (3 weeks of age)	Amide II (proteins)<br>-C=C- and -C=N (tryptophan, porphyrin, guanine, adenine)	FTIR, Raman
1645-1710 cm^{-1}	- in NOD vs control (3 weeks of age)	Amide I (proteins, mostly helical random coil structures)	FTIR

Table S3. OPLS-DA model details. Number of data points (spectra) are given as Total (N*B*C), where N is the number of spectra / biological replicate, B is the number of biological replicates / class, and C is the total number of classes (e.g. cell type, age, genotype, etc). Components are given as predictive + orthogonal, using C-1 predictive components to differentiate C classes, and 0 to C+1 orthogonal components for illustrating model evolution. The selected number of components for the final model are shown in bold and underlined (primarily based on cross-validated Q2(cum) values but never exceeding C orthogonal components to avoid potential overfitting, irrespective of improving Q2(cum) values. The number of components were kept constant for associated models to facilitate direct comparisons). R2X(cum) and R2Y(cum) stand for the cumulative fraction of the sum of squares of the entire X block (wavenumber variables) and Y-block (class variables), respectively, explained by all the components (i.e. explained fraction of X and Y variation, respectively). Q2(cum) stands for the cumulative fraction of the total variation of the Y block (class variables) that can be predicted by the components (i.e. the predictive ability of the model). All spectra that the models are based on are provided in Supplementary Dataset 1 (in Excel format).

Model	Number of data points (spectra)	Components	R2X(cum)	R2Y(cum)	Q2(cum)
Visualising cell type differences, FTIR spectra (Fig. 1)	20 (10 * 1 * 2)	1+0<br>1+1<br>**1+2**<br>1+3	0.620<br>0.865<br>**0.932**<br>0.959	0.784<br>0.885<br>**0.946**<br>0.977	0.763<br>0.824<br>**0.889**<br>0.923
Visualising cell type differences, Raman spectra (Fig. 1)	20 (10 * 1 * 2)	1+0<br>1+1<br>**1+2**<br>1+3	0.551<br>0.779<br>**0.855**<br>0.933	0.797<br>0.949<br>**0.975**<br>0.985	0.771<br>0.918<br>**0.931**<br>0.957
Visualising NOD cell type differences, FTIR spectra (Fig. S1)	30 (10 * 1 * 3)	2+0<br>2+1<br>2+2<br>**2+3**<br>2+4	0.912<br>0.939<br>0.983<br>**0.986**<br>0.990	0.445<br>0.626<br>0.722<br>**0.825**<br>0.867	0.382<br>0.473<br>0.617<br>**0.638**<br>0.666
NOD cell type differences, Raman spectra (Fig. S1)	30 (10 * 1 * 3)	2+0<br>2+1<br>2+2<br>**2+3**<br>2+4	0.861<br>0.941<br>0.959<br>**0.969**<br>0.980	0.304<br>0.377<br>0.446<br>**0.545**<br>0.591	0.141<br>0.144<br>-0.108<br>**-0.158**<br>-0.162
RIP-hIAPP amyloid plaque deposits in islets, FTIR spectra (Fig. S2)	25 (15/10 * 1 * 2)[1]	1+0<br>1+1<br>**1+2**<br>1+3	0.557<br>0.864<br>**0.945**<br>0.953	0.591<br>0.650<br>**0.694**<br>0.833	0.584<br>0.573<br>**0.576**<br>0.367
RIP-hIAPP amyloid plaque deposits in islets, Raman spectra (Fig. S2)	30 (15 * 1 * 2)	1+0<br>1+1<br>**1+2**<br>1+3	0.985<br>0.996<br>**0.997**<br>0.999	0.970<br>0.979<br>**0.986**<br>0.992	0.969<br>0.976<br>**0.976**<br>0.980
ob/ob vs control, endocrine, all ages, FTIR spectra (Fig. S4, Table S1, Movie S1)	60 (5 * 3 * 4)	3+0<br>3+1<br>**3+2**<br>3+3<br>3+4	0.828<br>0.874<br>**0.927**<br>0.947<br>0.967	0.292<br>0.444<br>**0.499**<br>0.543<br>0.583	0.226<br>0.334<br>**0.398**<br>0.371<br>0.435

		3+5	0.979	0.619	0.450
ob/ob vs control, endocrine, all ages, Raman spectra (Fig. S4, Table S1, Movie S1)	60 (5 * 3 * 4)	3+0<br>3+1<br>3+2<br>3+3<br>**3+4**<br>3+5	0.426<br>0.562<br>0.610<br>0.686<br>**0.712**<br>0.746	0.521<br>0.661<br>0.796<br>0.853<br>**0.911**<br>0.936	0.373<br>0.536<br>0.674<br>0.747<br>**0.817**<br>0.838
ob/ob vs control, exocrine, all ages, FTIR spectra (Fig. S4, Table S1, Movie S1)	60 (5 * 3 * 4)	3+0<br>3+1<br>**3+2**<br>3+3<br>3+4<br>3+5	0.823<br>0.908<br>**0.922**<br>0.952<br>0.962<br>0.968	0.328<br>0.380<br>**0.449**<br>0.502<br>0.543<br>0.609	0.243<br>0.296<br>**0.246**<br>0.232<br>0.195<br>0.165
ob/ob vs control, exocrine, all ages, Raman spectra (Fig. S4, Table S1, Movie S1)	60 (5 * 3 * 4)	3+0<br>3+1<br>3+2<br>3+3<br>**3+4**<br>3+5	0.516<br>0.589<br>0.622<br>0.655<br>**0.689**<br>0.712	0.476<br>0.617<br>0.749<br>0.831<br>**0.884**<br>0.910	0.374<br>0.478<br>0.576<br>0.606<br>**0.643**<br>0.675
ob/ob vs control, endocrine, 9 weeks only, FTIR spectra (Fig. 2, Table S1)	30 (5 * 3 * 2)	1+0<br>1+1<br>**1+2**<br>1+3	0.420<br>0.631<br>**0.828**<br>0.880	0.599<br>0.792<br>**0.846**<br>0.918	0.557<br>0.718<br>**0.792**<br>0.846
ob/ob vs control, endocrine, 9 weeks only, Raman spectra (Fig. 2, Table S1)	30 (5 * 3 * 2)	1+0<br>1+1<br>**1+2**<br>1+3	0.243<br>0.506<br>**0.605**<br>0.696	0.443<br>0.701<br>**0.904**<br>0.961	0.166<br>0.497<br>**0.779**<br>0.847
ob/ob vs control, exocrine, 9 weeks only, FTIR spectra (Fig. 2, Table S1)	30 (5 * 3 * 2)	1+0<br>1+1<br>**1+2**<br>1+3	0.517<br>0.728<br>**0.769**<br>0.824	0.446<br>0.784<br>**0.914**<br>0.933	0.397<br>0.704<br>**0.804**<br>0.864
ob/ob vs control, exocrine, 9 weeks only, Raman spectra (Fig. 2, Table S1)	30 (5 * 3 * 2)	1+0<br>1+1<br>**1+2**<br>1+3	0.459<br>0.562<br>**0.679**<br>0.729	0.345<br>0.763<br>**0.870**<br>0.959	0.268<br>0.545<br>**0.655**<br>0.739
NOD vs control, endocrine, FTIR spectra (Fig. 3, Table S2, Movie S2)	90 (5 * 3 * 6)	5+0<br>5+1<br>5+2<br>5+3<br>5+4<br>**5+5**<br>5+6	0.928<br>0.946<br>0.968<br>0.974<br>0.980<br>**0.983**<br>0.986	0.489<br>0.582<br>0.615<br>0.651<br>0.688<br>**0.720**<br>0.752	0.430<br>0.490<br>0.522<br>0.547<br>0.573<br>**0.593**<br>0.591
NOD vs control, endocrine, Raman spectra (Fig. 3, Table S2, Movie S2)	90 (5 * 3 * 6)	5+0<br>5+1<br>5+2<br>5+3<br>5+4<br>**5+5**<br>5+6	0.846<br>0.858<br>0.873<br>0.883<br>0.889<br>**0.897**<br>0.905	0.726<br>0.778<br>0.829<br>0.843<br>0.887<br>**0.899**<br>0.916	0.660<br>0.703<br>0.755<br>0.764<br>0.796<br>**0.809**<br>0.829
NOD vs control, exocrine, FTIR spectra	90 (5 * 3 * 6)	5+0<br>5+1	0.913<br>0.938	0.443<br>0.513	0.387<br>0.458

(Fig. 3, Table S2, Movie S2)		5+2<br>5+3<br>5+4<br>**5+5**<br>5+6	0.949<br>0.960<br>0.964<br>**0.969**<br>0.976	0.545<br>0.597<br>0.670<br>**0.693**<br>0.709	0.463<br>0.481<br>0.547<br>**0.530**<br>0.543
NOD vs control, exocrine, Raman spectra (Fig. 3, Table S2, Movie S2)	90 (5 * 3 * 6)	5+0<br>5+1<br>5+2<br>5+3<br>5+4<br>**5+5**<br>5+6	0.798<br>0.816<br>0.837<br>0.849<br>0.862<br>**0.876**<br>0.882	0.647<br>0.728<br>0.798<br>0.841<br>0.861<br>**0.877**<br>0.900	0.566<br>0.631<br>0.701<br>0.736<br>0.757<br>**0.771**<br>0.781
Visualising transplanted islets, RACE (Fig. 4)	20 (10 * 1 * 2)	1+0<br>1+1<br>**1+2**<br>1+3	0.678<br>0.976<br>**0.989**<br>1.000	0.880<br>0.970<br>**0.982**<br>0.984	0.870<br>0.962<br>**0.970**<br>0.974
Visualising cell types through the tissue capsule, Raman spectra (Fig. 5)	20 (10 * 1 * 2)	1+0<br>1+1<br>**1+2**<br>1+3	0.970<br>0.986<br>**0.989**<br>0.993	0.982<br>0.984<br>**0.987**<br>0.989	0.981<br>0.979<br>**0.959**<br>0.949
NOD vs control proof of concept, endocrine, intact pancreata, Raman spectra (Fig. 5)	25 (5 * 2/3 * 2)[2]	1+0<br>1+1<br>**1+2**<br>1+3	0.919<br>0.936<br>**0.970**<br>0.973	0.943<br>0.967<br>**0.976**<br>0.986	0.931<br>0.914<br>**0.932**<br>0.966
NOD vs control proof of concept, exocrine, intact pancreata, Raman spectra (Fig. 5)	25 (5 * 2/3 * 2)[2]	1+0<br>1+1<br>**1+2**<br>1+3	0.791<br>0.852<br>**0.993**<br>0.995	0.937<br>0.965<br>**0.973**<br>0.987	0.933<br>0.961<br>**0.967**<br>0.972

[1] Due to diffraction limits in the spatial resolution of FTIR microspectroscopy, only the largest plaques could be used, hence the varying number of spectra ("15/10")
[2] Biological replicates "2/3" refer to 2 NOD and 3 NOD.H2b (control) mice.

Nord et al., Movie S1 (Still)

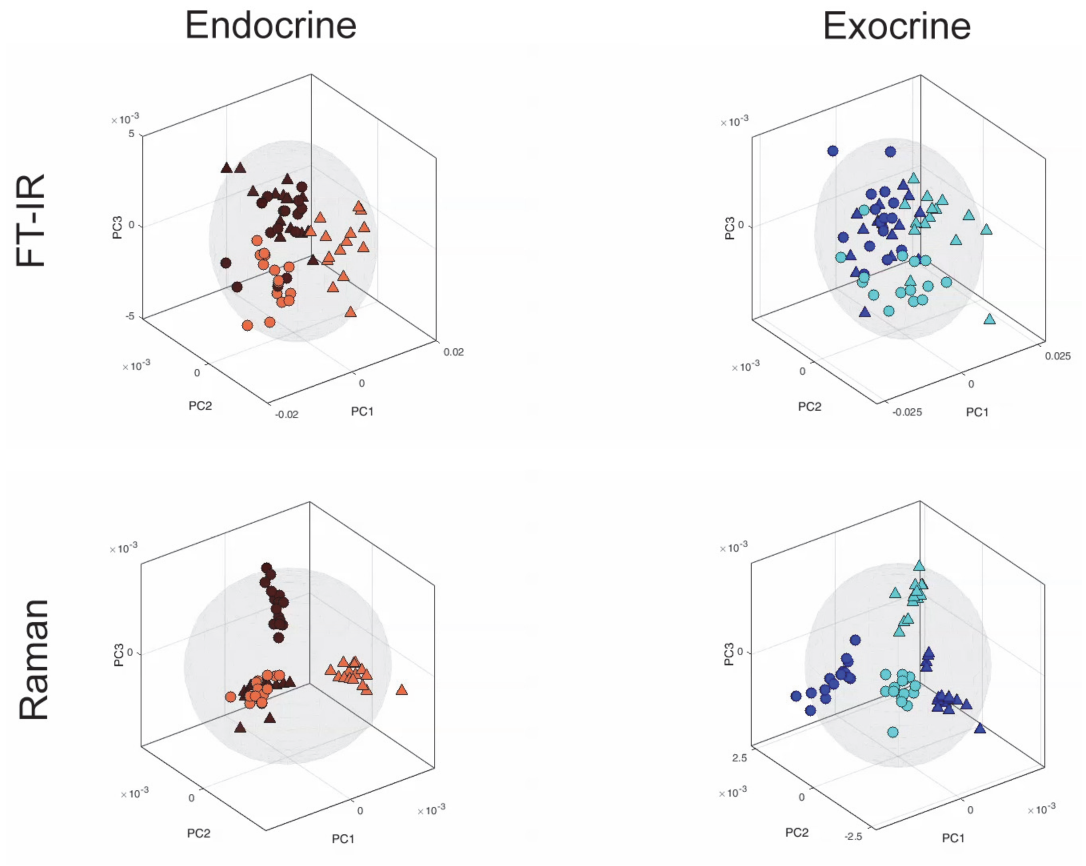

Movie S1. 3D OPLS-DA Scores plots based on FTIR and Raman spectra of endocrine and exocrine regions from 3 and 9 weeks old *ob/ob* and *+/?* control mice. Circles represent *ob/ob* mice and triangles represent control mice. Light shading denotes 3-week-old individuals and dark shading 9-week old individuals. The ellipsoid corresponds to the 95% confidence interval of the model (Hotellings T2).

Nord et al., Movie S2 (Still)

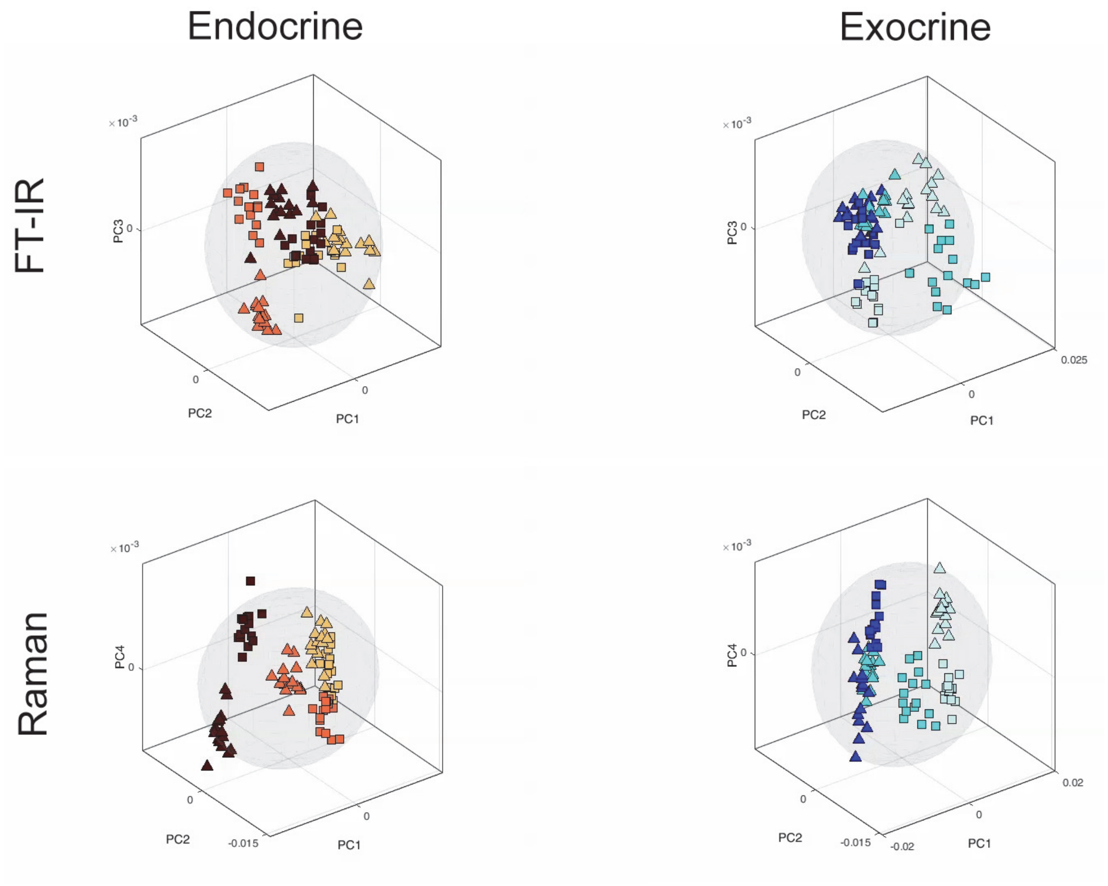

Movie S2. 3D OPLS-DA Scores plots based on FTIR and Raman spectra of endocrine and exocrine regions from 1, 3 and 9 weeks old NOD and NOD.H2-b control mice. Squares represent NOD mice and triangles represent NOD.H2-b control mice. The lightest shading denotes 1-week, medium shading 3-week and darkest shading 9-week old individuals. The ellipsoid corresponds to the 95% confidence interval of the model (Hotellings T2).

Supplementary Dataset 1. FT-IR and Raman spectra used for the presented models.

Each sheet contains one set of data (FT-IR or Raman) for one model. The model to which the spectra belong are described in the sheet titles and the figure in which the model is shown is listed in the first (A1) cell of each sheet with yellow background. Model details are listed in Supplementary Table 3.

The second row of each worksheet contains the wavenumbers for the spectra (X variables). Thereafter, each row is one spectrum, with the first column being the observation identifier (name) of the spectrum. Spectrum names contain the class information as described in the "Notations" sheet of the dataset: the first characters describing cell type, thereafter genotype, age (when relevant) and other identifiers. The names always end with the pixel number depicting the position from which the spectrum was extracted from a hyperspectral image to provide a unique identifier for spectra from the same image and cell types. All spectra are pre-treated as described in the Materials and Methods section.
